# Environmental Impacts of Kapok–Cotton Seed Fibers and Waste Tire Rubber Powder-Incorporated Hybrid Mortar System: A Cradle-to-Gate Life Cycle Assessment

**DOI:** 10.3390/polym18202527

**Published:** 2026-10-15

**Authors:** Gülşah Susurluk

**Affiliations:** Textile Technology Department, School of Advanced Vocational Studies, Istanbul Beykent University, Istanbul 34520, Turkey; gulsahsusurluk@beykent.edu.tr

**Keywords:** kapok–cotton seed fibers, waste tire rubber powder, life cycle assessment, environmental impact

## Abstract

Owing to their well-established formulations and high performance, traditional cement-based materials are among the most widely used construction materials worldwide. However, their production generates substantial greenhouse gas emissions and relies heavily on the unsustainable consumption of limited natural resources. Increasingly stringent environmental regulations have therefore made reducing their environmental footprint a priority for both industrial sector and academic institutions. This study investigated the effects of incorporating kapok–cotton seed fibers (KCSFs) and waste tire rubber powder (WTRP) into conventional cement mortars on their mechanical properties, thermal conductivity, and life cycle environmental impacts. For this purpose, five mortar mixtures containing different content ratios of KCSFs and WTRP were prepared. Their mechanical properties, thermal conductivity, microstructure, mineralogical and pore characteristics were comprehensively evaluated, together with a comparative life cycle assessment (LCA). The results showed that all mixtures maintained 28-day compressive strengths above 40 MPa, while flexural strength increased by up to approximately 18% and thermal conductivity decreased by up to approximately 32% compared with the reference mortar. Moreover, the HybTP-0.5 mixture demonstrated an approximately 8.5% lower global warming potential (GWP) than the reference mortar. However, environmental trade-offs were observed, with the Hyb-0.5 mixture exhibiting increases of approximately 118% in eutrophication potential (EP) and 19% in terrestrial ecotoxicity (TE), while the corresponding increases for HybTP-0.5 were approximately 115% and 8%, respectively. These findings indicate that the environmental benefits varied across impact categories and mixture compositions. Overall, the findings indicate that the combined incorporation of KCSFs and WTRP improved flexural strength and reduced thermal conductivity while maintaining compressive strengths above 40 MPa, although the environmental outcomes varied across impact categories. These results highlight the potential of incorporating bio-based and waste-derived constituents into conventional cement mortars without requiring substantial modifications to established mixture-design and production practices.

## 1. Introduction

### 1.1. Background

The increasing global energy demand, the rise in the rate of construction, and international goals to combat climate change have made it imperative to reassess the environmental impact of materials utilized in the construction industry. Cement-based materials such as concrete and mortar are among the most consumed materials in the world after water, with annual production reaching approximately twenty-five billion tons [1]. This widespread utilization leads to serious environmental problems due to the high energy requirements of the cement production process and the CO_2_ emissions released during the calcination of limestone [2,3,4,5]. Today, the construction industry is reportedly responsible for between 30 and 40% of global energy consumption and up to 40% of greenhouse gas emissions [6]. Considering these environmental challenges, minimizing the carbon footprint of construction materials, restricting the utilization of natural resources, and enhancing functional performance have emerged as an essential research priority. According to the Paris Climate Agreement, the European Union, via the European Green Deal and the Fit for 55 policy package, aims to diminish greenhouse gas emissions by a minimum of 55% by 2030 and achieve climate neutrality by 2050 [7,8,9,10,11,12]. These goals encourage the development of solutions that focus on low-carbon and circular economy principles, especially in the industry of building materials [13,14].

In this context, reducing the cement content in cement-based systems and partially replacing it with supplementary cementitious materials or natural and waste-derived constituents has become a key strategy for improving sustainability. In recent years, the incorporation of industrial waste and natural fibers into mortar and concrete systems has been regarded as an effective approach for reducing environmental consequences while enhancing mechanical, thermal, and durability properties. Natural fibers are attracting interest in cement-based composites because of their renewable nature, low density, ability to prevent fracture propagation, potential to decrease thermal conductivity, and enhancement of flexural strength [15,16].

Plant seed fibers, classified as natural fibers, possess the capacity to diminish density and affect thermal transfer mechanisms in cement-based composites owing to their hollow or semi-hollow lumen structures and low specific gravity. The porous structure generated by the fibers in the matrix enhances thermal insulation efficacy by restricting conduction and convection heat-transfer mechanisms [16,17]. Kapok (KP) and cotton (CO) fibers, both derived from seeds, exhibit considerable differences in their morphological, physical, and surface chemical characteristics, and perform different functional roles in cement-based systems. Kapok fiber stands out as a lightweight addition in construction materials owing to its extremely low density (≈0.29 g/cm^3^), a void ratio that can range from 80 to 90%, and an air-filled lumen structure, making it a biomaterial with significant potential for decreasing thermal conductivity [18,19,20,21,22]. The fiber’s largely air-filled internal structure interrupts the continuous phase inside the matrix, restricting heat transfer; simultaneously, it forms a microstructure that enhances acoustic and thermal insulation efficacy by increasing total porosity [16]. However, the hydrophobic surface and restricted chemical reactivity of kapok fiber may result in inadequate mechanical bonding at the fiber–matrix interface. On the other hand, cotton fiber, characterized by its elevated cellulose content, rough surface morphology consisting of microfibrils, and natural surface constituents (wax, hemicellulose, and lignin), supports enhanced mechanical interlocking with the cement matrix. Research indicates that cotton fibers limit crack development via a fiber bridging mechanism and enhance mechanical performance parameters, including flexural strength [15,23]. Experimental findings indicate that wax and other non-cellular constituents on cotton fiber surfaces significantly influence fiber-to-fiber and fiber-to-matrix friction, with this surface chemistry being crucial in bonding and energy absorption mechanisms [24]. Furthermore, the fact that cotton fiber has a relatively low thermal conductivity value among natural fibers indicates that it can also contribute to thermal performance when utilized in appropriate proportions [17].

In addition to natural fiber reinforcement, the use of WTRP in cement-based systems within the framework of the circular economy approach has also gained increasing research interest in recent years. Global end-of-life tire generation is estimated at around 1.5 billion units per year, reflecting the accelerating scale of tire waste worldwide [25,26]. The storage and disposal of these wastes provide considerable challenges regarding land utilization, fire hazards, and environmental pollution; thus, incorporating them into building materials provides a substantial and sustainable recycling method. Literature reports indicate that the inclusion of fine-fraction tire rubber powders in cement-based mortar and concrete systems reduces unit weight due to their low specific gravity and elastic structure, and weakens heat-transfer mechanisms by forming a discontinuous phase with low thermal conductivity within the matrix [27,28,29]. The porous structure and high Poisson ratio of rubber particles diminish the solid–solid contact area, hence restricting heat transmission and reducing impact and vibrational energy [1]. At the same time, the hydrophobic characteristics of waste tire dust and its restricted interfacial interaction with the cement matrix may cause void formation, especially in the interfacial transition zone (ITZ), hence diminishing mechanical properties such as compressive and flexural strength. Many studies have shown that strength losses become more pronounced with increasing tire rubber powder content; this is primarily related to weak bonding, low modulus of elasticity, and stress concentration [1,28]. However, it is underlined that systems utilizing WTRP demonstrate enhanced deformation capacity and energy absorption characteristics; thus, tire rubber powder should be regarded as a component that diminishes rigidity while increasing ductility.

Recent research indicates that mechanical weaknesses resulting from tire dust can be overcome with fiber reinforcement. In particular, the crack-bridging mechanism of the fibers and their capacity to sustain load transfer under tension can partially mitigate the weak interfacial regions formed by tire rubber powder, hence enhancing the residual strength and deformation performance of the composite. In this context, the comprehensive evaluation of the weight-reducing and thermal insulation-enhancing properties of rubber dust, with the mechanical and microstructural properties of natural fibers, facilitates a more balanced and multifunctional performance than the use of different additives [29,30,31]. In this context, the comprehensive evaluation of the weight-reducing and thermal insulation-enhancing properties of rubber dust, with the mechanical and microstructural properties of natural fibers, facilitates a more balanced and multifunctional performance than the use of different additives. However, current work predominantly emphasizes synthetic or metallic fibers at the cement-based mortar systems; investigations into these systems using both plant-based KCSFs and WTRP are extremely limited.

### 1.2. Literature Review

In recent years, the incorporation of natural fibers and by-products, such as recycled WTRP, in cement-based composites has attracted increased attention in terms of environmental sustainability and functional performance. Research on the inclusion of natural fibers into cementitious systems has indicated enhancements, especially in flexural strength, attributed to the fiber bridging mechanism. Anju et al. [15] reported an enhancement in flexural strength and a delay in fracture propagation in mortar systems with 0.5–1.0% cotton fiber. Wongsa et al. [32] indicated that including natural fibers at 0.25–0.75% enhanced flexural performance; however, increased fiber content resulted in diminished inability and compressive strength. At the same time, investigations indicate that enhancements in flexural strength and energy absorption capacity can be achieved based on cotton fiber content; nevertheless, mechanical improvements are limited if the optimum fiber ratio is exceeded [23,24]. Research on kapok fiber indicates that it is primarily used as a lightweight and thermal insulation addition in cementitious systems. Yang et al. [19] and Sun et al. [21] highlighted that the thermal performance of cement-based systems is enhanced by the incorporation of kapok fiber; however, the mechanical contribution is limited to the interaction between the fiber and the matrix. Similarly, Susurluk et al. [24] indicated that significant reductions in thermal conductivity were achieved in cement-based systems with a kapok fiber content ratio of 0.5–1.0%, although enhancements in flexural and compressive strength were limited. Wijesinghe et al. [16,17] demonstrated that in cement-based mortars reinforced by different natural and textile fibers, the fiber content modifies the pore structure and void distribution, enhancing thermal conductivity and sound absorption performance; however, excessive fiber content ratios may adversely affect mechanical strength.

The investigation focuses on the incorporation of WTRP in cement-based composites, examining its weight-loss effect attributed to its elastic properties and low specific gravity, as well as its influence on thermal conductivity. Eldin and Senouci [33] and Topcu [34] indicated that in cement-based systems utilizing rubber as a substitute for aggregate at a rate of 10–30%, the unit volume weight diminishes; nonetheless, notable reductions in compressive and flexural strengths are observed. Segre and Joekes [35] demonstrated at the microstructural level that poor adhesion between tire rubber particles and the cement matrix is the primary cause of reductions in mechanical strength. Cairns et al. [36] reported that the unit weight and compressive strength were reduced in cement-based systems containing recycled tire rubber aggregate. Fadiel et al. [37] indicated that substituting 10–40% of sand with rubber powder diminished thermal conductivity by 13–21%, but significant reductions in flexural deformation were observed. Zhang et al. [28] highlighted that in systems with 15% or more rubber powder, significant reductions in flexural strength occurred due to poor bonding at the matrix–rubber interaction. Liu et al. [38] and Hancharoen et al. [39] similarly indicated that in combinations with tire rubber powder including 5–30%, thermal conductivity could decrease by 10–40%; nevertheless, an increase in content ratio resulted in a reduction of flexural strength by 15–50%.

Literature findings indicate that lightweight and elastic additions, such as waste tire rubber powder, enhance insulation performance by reducing thermal conductivity; however, these improvements are generally associated with reductions in flexural strength. Similarly, although the individual utilization of natural fibers provides a positive impact on mechanical performance and durability, the assessment of these effects in relation to thermal insulation lightweight systems has been limited. This situation highlights the significance for complementary and synergistic strategies that enhance thermal and mechanical performance simultaneously. This approach utilizes the combined properties of KCSFs in cement-based green mortar systems with waste tire rubber powder, utilizing the low density and thermal insulation of kapok to enhance the mechanical performance and flexural behavior provided by cotton fiber in a synergistic manner. The lack of research in the existing literature that concurrently examine these three components and assess thermal-mechanical performance comprehensively requires an extensive investigation into the potential of such hybrid mortar systems.

### 1.3. Research Significance

The fundamental approach of this study is to improve the mechanical, thermal, and environmental performance of traditional cement mortars by incorporating kapok–cotton fibers and WTRP, while maintaining the mechanical performance and easy production process used traditional Portland cement-based mortar structure. In this context, the aim was to limit mechanical losses linked to WTRP utilization and to achieve a balance between mechanical and thermal performance by combining the contribution of kapok fiber’s low-density, hollow structure to thermal performance with the crack-bridging potential of cotton fiber. In this study, kapok and cotton fibers were used together at a fixed 1:1 ratio as a combined seed fiber system. Accordingly, the present study focuses on the overall performance of this predefined fiber system with and without WTRP, while the individual contributions and interaction effects of the constituents are beyond the scope of the current experimental design. The novelty of this study demonstrated not only in evaluating the influence of these components on mechanical and thermal characteristics but also in the comprehensive assessment of the relationships among performance, microstructure, and environmental impact. Mechanical and thermal properties were evaluated together with microstructural, mineralogical, and pore characteristics, while exploratory regression analyses were used to visualize trends among the measured properties. Furthermore, the environmental performance of the developed mortars was assessed through a cradle-to-gate LCA. Thus, while maintaining the traditional Portland cement-based mortar structure and practical production approach, this study presents a holistic material design approach based on the combined incorporation of bio-based and waste-derived components, with the aim of maintaining mechanical performance while improving thermal performance and reducing environmental impacts in selected life cycle impact categories. This approach is consistent with the broader objectives of resource efficiency, circularity, and greenhouse gas emission reduction promoted by the European Green Deal and the “Fit for 55” framework.

## 2. Materials and Methods

### 2.1. Materials

This study utilized CEM I 42.5 R type Portland cement, compliant with the TS EN 197-1 [40] standard, supplied from Afyon Cement as the binder material. This cement consists of only Portland cement clinker and gypsum, and its high early strength renders it the preferable binder in mortar systems, supporting rapid hydration development. The Portland cement utilized includes a chemical composition that facilitates early strength development, containing roughly 63% CaO. Additionally, the contents of SiO_2_ (≈19%), Al_2_O_3_ (≈5%), and Fe_2_O_3_ (≈3%) indicate that the cement displays conventional mineralogical properties of Portland cement. The contents of MgO (≈2%), SO_3_ (≈3%), and chloride (≈0.03%) conform with the TS EN 197-1 [40] standard limits. In terms of physical properties, the cement used has a specific surface area of approximately 4150 cm^2^/g and a specific gravity of 3.11 g/cm^3^, supporting for rapid initiation of hydration reactions upon contact with water. These qualities enhance the interaction between binders and additives in mortar systems via fiber and waste-based additives.

This study utilized standard Rilem-Cembureau-type sand, provided by Trakya Limak Cement Factory in Kırklareli, as fine aggregate. The sand was generated in compliance with the TS EN 196-1 [41] standard and selected to provide repeatability and comparability in experimental studies regarding grain size distribution and physical qualities. Accordingly, homogeneous and standardized sand was utilized in all mixes to accurately assess the mechanical and thermal properties of the mortar mixtures.

Furthermore, waste tire rubber powder, an industrial byproduct, was evaluated as a partial substitute material in the study. Waste tire rubber powder with a particle size of 0–0.5 mm, provided by Katek Ağaoğlu Rubber Technologies (Uşak, Turkey), was utilized as an addition to diminish thermal conductivity in mortar systems.

This study utilized kapok and cotton fibers as natural fiber additions. Kapok fiber was evaluated as a biomaterial that enhances thermal insulation efficacy in mortar systems, attributed to its low density (≈0.29 g/cm^3^), elevated void ratio, and low thermal conductivity ranging from 0.04 to 0.06 W/mK. Cotton fiber, displaying a density of approximately 1.50–1.55 g/cm^3^, a fiber diameter of 10–20 µm, and a tensile strength ranging from 300 to 600 MPa, serves as a natural fiber that enhances flexural behavior, particularly via a fiber bridging mechanism, attributable to its cellulose-based structure. The use of both fibers aims to achieve multifunctional and sustainable performance in cement-based mortar systems by synergistically integrating the lightweight and thermal insulation properties of kapok fiber with the mechanical performance-enhancing capabilities of cotton fiber.

Associated with the C_3_S and C_2_S phases, indicating the reactive phase content of the binder system. In the slag sample, a broad amorphous hump was detected in the range of 20–35° 2θ, rather than a definite crystalline peak, indicating that the slag was mainly amorphous and possessed a high reactivity potential. In the literature, it is known that amorphous slag dissolves during hydration and/or alkali activation processes, contributing to the formation of mainly calcium silicate hydrate (C–S–H) and aluminum-containing calcium silicate hydrate (C-(A)-S-H) gel phases. It is stated that these gel phases also significantly contribute to the improvement of mechanical and strength properties by increasing the density of the matrix [22]. The XRD pattern of kapok fiber exhibited broad, low-intensity amorphous regions rather than identifiable crystalline peaks, indicating the semi-crystalline character of fibers with a lignocellulosic structure. Similarly, the WTRP sample also exhibits low crystallinity and a broad amorphous distribution, a structure associated with the presence of elastomeric components. It is known that these types of amorphous admixtures create filler and weak interfacial effects on the microstructure rather than directly forming a crystalline phase in the cement matrix. Prominent and intense peaks were detected in the lithium carbonate sample, signifying a high level of crystallinity in the material. This crystalline structure can create possible nucleation centers that may influence the reaction kinetics within the system. The broad diffraction peak at around 25° (2θ) in the PC sample, along with the broad and low-intensity diffraction features in the 40–45° range, signifies that the material is primarily amorphous carbon and displays an irregular microstructure with a low degree of crystallinity.

### 2.2. Design and Proportions of the Mixtures

The current research focuses on involving the design of five different mortar mixtures to create sustainable mortars that enhance thermal insulation performance while maintaining sufficient flexural strength. Thus, in mix designs, a natural fiber system including WTRP and kapok and cotton fibers was assessed together. All mixtures were formulated using drinkable tap water; the water/cement ratio was kept at 1:2 and the cement/sand ratio at 1:3. To enhance thermal performance, WTRP was incorporated as a 10% mass-based replacement of natural sand in two mixtures. Owing to the lower density of WTRP relative to natural sand, this substitution resulted in a higher volumetric contribution of the rubber phase within the aggregate system. Accordingly, the WTRP replacement level throughout this study is expressed on a mass basis. Cotton and kapok fibers were incorporated into the mixes as a seed fiber system. The fiber content was defined relative to the mass of cement. Kapok and cotton fibers were incorporated at an equal mass-based ratio (1:1), resulting in total fiber contents of 0.5% and 1.0% by cement mass (0.25% kapok + 0.25% cotton and 0.5% kapok + 0.5% cotton, respectively). Because the two fiber types have different physical characteristics and densities, the equal mass-based ratio should not be interpreted as representing equal volumetric proportions. Fiber dosages were defined on a cement-mass basis throughout this study; accordingly, volumetric fiber fractions were not considered as a mixture-design parameter. The control mixture, lacking of fiber and WTRP, is designated by the code Hyb-0. Mixes containing KCSFs are designated as Hyb-0.25 and Hyb-0.5, depending upon their fiber content. The experimental matrix was designed to evaluate the combined performance of the KCSF system and WTRP; therefore, individual constituent effects and statistical interaction effects were not independently quantified. Mixtures comprising WTRP with same fiber ratios are designated as HybTP-0.25 and HybTP-0.5, respectively. All mixture mortar designs and proportions are presented in Table 1.

### 2.3. Preparation of Mortar Specimens and Experimental Program

In the current investigation, each raw material was measured with a precision balance, and mortar mixtures were prepared using a Hobart-type laboratory mixer. Initially, Portland cement and sand were mixed dry, then tap water was added in specific proportions to obtain a homogeneous mortar matrix. Kapok and cotton fibers, used as natural fiber additives, were added to the mixture after the fresh mortar matrix had reached sufficient workability, in accordance with the procedure described in the literature as the “after mixing method” and recommended by Gao et al. [42]. This approach was implemented to prevent fiber clumbing and ensure a homogeneous distribution of fibers within the matrix [43]. The nominal water-to-cement ratio was maintained at 0.50 for all mixtures, with no additional water adjustment to compensate for fiber water uptake. No superplasticizer or other chemical admixture was used in the mixtures.

The prepared fresh mortars were placed into prismatic steel molds of 40 × 40 × 160 mm, in compliance with the TS EN 196-1 [41] standard, and compressed using mechanical vibration. The samples were retained in their molds under laboratory conditions of 21 ± 1 °C for the initial 24 h, thereafter removed from the molds, and cured in water at the same temperature until testing was conducted. Flexural strength tests were conducted at cured for 7 and 28 days utilizing the three-point flexural test method in compliance with the TS EN 1015-11 [44] standard. Three replicate specimens were tested for each mixture, and the mechanical strength results were reported as mean values with their corresponding standard deviations. The residual parts obtained from the samples that fractured after the flexural test were utilized in compressive strength, thermal conductivity, porosity, microstructure and mineralogical analysis. The thermal conductivity coefficient (W/m·K) of the mortar samples was measured using a C-THERM/TCi Thermal Conductivity Analyzer (C-Therm Technologies Ltd., Fredericton, NB, Canada) employing the Modified Transient Plane Source (MTPS) technique, which has previously been applied to the thermal characterization of cement-based mortars [45]. Prior to testing, the 28-day specimens obtained from the mechanical strength tests were oven-dried at 50 ± 5 °C for 24 h to reduce their moisture content and establish a consistent preconditioning procedure. The specimens were subsequently tested at a room temperature of 21 ± 1 °C. However, the attainment of a constant-mass condition was not independently verified. Measurements were repeated five times on different specimen faces, and the thermal conductivity coefficient was reported as the arithmetic mean of five individual measurements. Microstructural analyses were conducted on fragments taken from the fracture surfaces of the samples utilizing a Phenom ProX desktop scanning electron microscopy (SEM) (Phenom-World, Eindhoven, The Netherlands) located in the laboratories of the Uşak University Scientific Analysis and Technological Application and Research Center (UBATAM). Surface morphology, fiber–matrix interaction, porous structure and distribution of hydration products were evaluated using SEM images. For the purpose of mineralogical characterization, samples that underwent 28 days of curing were ground up in an agate mortar, passed through a 63 µm sieve, and examined with CuKα radiation within the 10–90° (2θ) scanning range utilizing a Rigaku MiniFlex XRD (Rigaku Corporation, Tokyo, Japan) instrument. XRD analyses were performed in UBATAM laboratories. BET (Micromeritics Instrument Corporation, Norcross, GA, USA) analyses were performed using a Micromeritics Gemini VII 2390t surface area analyzer at the Afyon Kocatepe University Technology Application and Research Center (TUAM), with N_2_ as the adsorptive. The specific surface area, total pore volume, and pore-size distribution of the mortar specimens were evaluated. Prior to analysis, the specimens were subjected to a two-stage degassing procedure at 90 °C for 60 min, followed by 350 °C for 240 min, with a heating rate of 10 °C/min. One specimen from each mortar mixture was analyzed; therefore, the BET results were used for descriptive and comparative microstructural assessment rather than statistical inference. It should be noted that the degassing procedure at 350 °C for 240 min may have affected the natural fibers, waste tire rubber powder, and cement hydration products, including C–S–H phases, potentially influencing the measured BET parameters. Therefore, the BET results should be interpreted with caution, as they may not fully represent the original pore structure of the mortar specimens. In addition to the experimental characterization, a life cycle assessment (LCA) was conducted to evaluate the environmental performance of the five mortar mixtures in accordance with ISO 14040 [46] and ISO 14044 [47], following four stages: goal and scope definition, life cycle inventory (LCI), life cycle impact assessment (LCIA), and interpretation. A cradle-to-gate approach was adopted, with a functional unit of 1 m^3^ of mortar. Raw material procurement, transportation, and electrical energy consumption during mortar production were included within the system boundaries. The mortar densities used for the life cycle inventory were assumed values adopted for theoretical calculations rather than experimentally measured densities. The laboratory-scale mixture quantities were converted to kg/m^3^ using these assumed densities while maintaining the mass proportions of the constituent materials in each mixture. The resulting material quantities used in the life cycle inventory are presented in Table 2.

This approach enabled the modeling of all material, energy, and transport inputs based on a common functional unit and ensured the comparability of different mortar mixtures. When incorporating waste tire rubber powder (WTRP) into the life cycle model, a representative “dummy product” was created in SimaPro because the database used did not contain a suitable product or production process that directly represented the specific WTRP utilized in the study. No higher-level environmental impact from its previously mentioned life cycle has been assigned to the waste tire; instead, the energy utilized in the shredding, crushing, grinding, granulation, and size classification processes necessary for transforming the waste tire into usable rubber powder has been incorporated within the system boundaries. In this context, an electrical energy consumption of 240 kWh/t was defined for WTRP production, and this energy input was allocated proportionally to the life cycle inventory based on the actual amount of WTRP used in each mixture. The assumed WTRP processing energy represents the processing stage only and does not include transportation. Transportation of WTRP was modeled separately using a road transport distance of 15 km, consistent with the material-specific transportation inventory. Thus, while preventing the environmental burden of WTRP from being considered entirely zero given that it is a recovered material the processing energy required to convert waste tires into a usable powder form has also been factored into the assessment.

The actual distances from supply sources to the mortar production facility were utilized to assess the environmental impact of raw material transportation. Transport inputs were calculated in tonne-kilometers (tkm), and the quantity of each raw material (in tonnes) within 1 m^3^ of mortar was correlated with the respective transport distance and incorporated into the life cycle inventory. Since the kapok fiber is sourced from the Philippines, the transportation process for this material has been modeled in three distinct stages: road transport within the Philippines, maritime transport between the Philippines and Turkey, and road transport within Turkey. The supplier locations, transportation methods, and distances utilized in the life cycle inventory are detailed in Table 3.

Road transportation was modeled utilizing the “Freight lorry 16–32 metric ton, EURO 4” methodology from the SimaPro database, indicating of medium-to-heavy load transportation. The kapok fiber transported via maritime routes from the Philippines to Turkey utilized the “Freight, sea, container ship” methodology. Thus, the environmental impacts that result from road and maritime transportation were integrated into the model through methods tailored to each transport mode.

The electrical energy utilized in mixing and mechanical vibration processes has been incorporated into the life cycle inventory concerning energy consumption involving mortar production. The energy required for production was determined using a functional unit of 1 m^3^ of mortar, with the energy inputs for each mixture detailed in Table 4.

**Figure 1:**
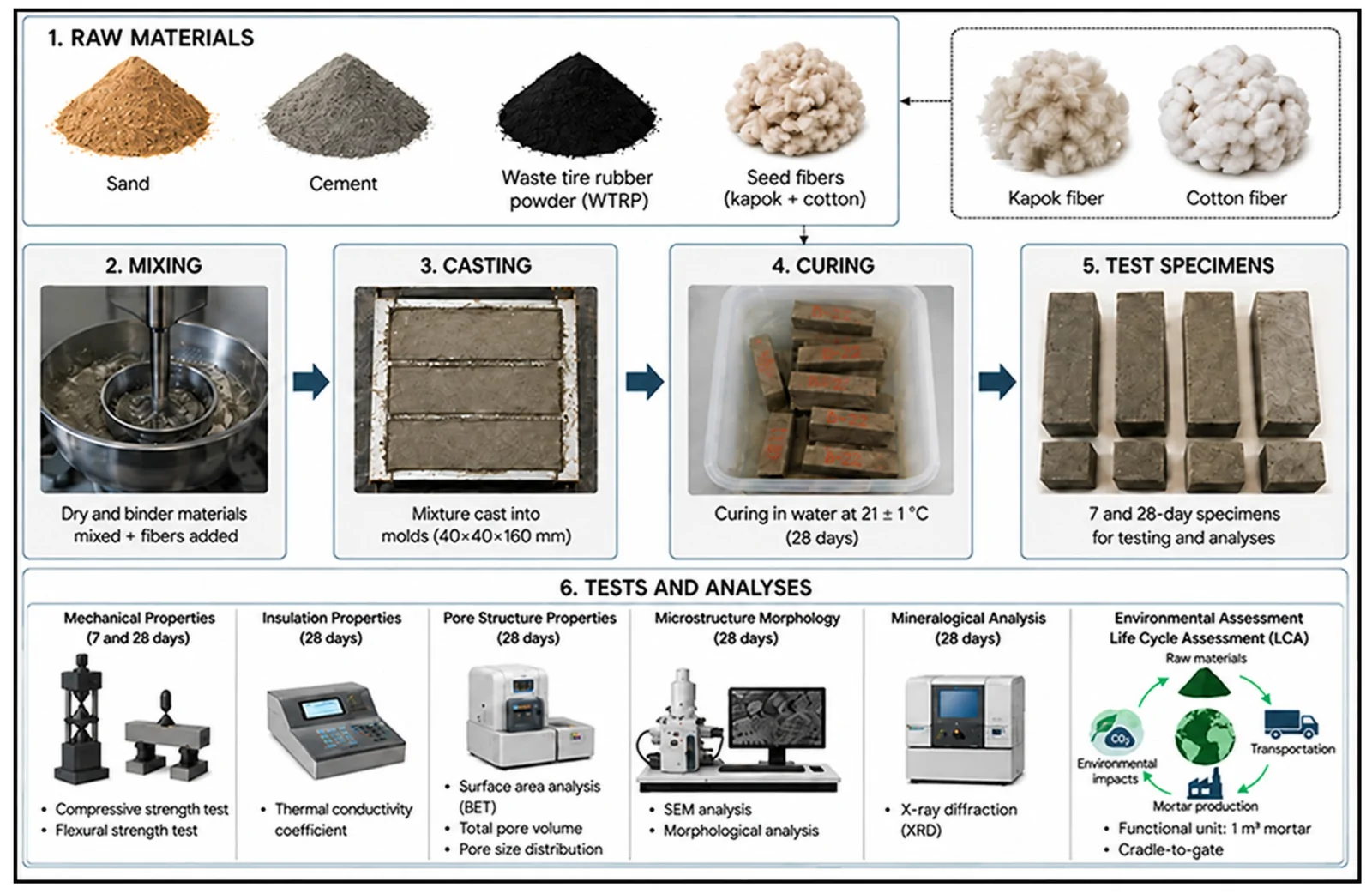
The general methodological framework of the study, comprising the mortar production process, experimental characterization and life cycle assessment.

The environmental impact assessment was conducted using SimaPro 9.5 software according to the CML-IA Baseline method. In that framework, 11 categories of environmental impact were assessed: abiotic depletion (AD), abiotic depletion–fossil fuels (AD-FF), global warming potential (GWP), ozone depletion potential (ODP), human toxicity (HT), freshwater aquatic ecotoxicity (FAE), marine aquatic ecotoxicity (MAE), terrestrial ecotoxicity (TE), photochemical oxidation (PO), acidification potential (AP), and eutrophication potential (EP). The general methodological framework of the research, which includes stages of mortar manufacture, experimental characterization methods and life cycle assessment is illustrated schematically in Figure 1.

## 3. Results and Discussion

### 3.1. Strength-Thermal Conductivity

**Figure 2:**
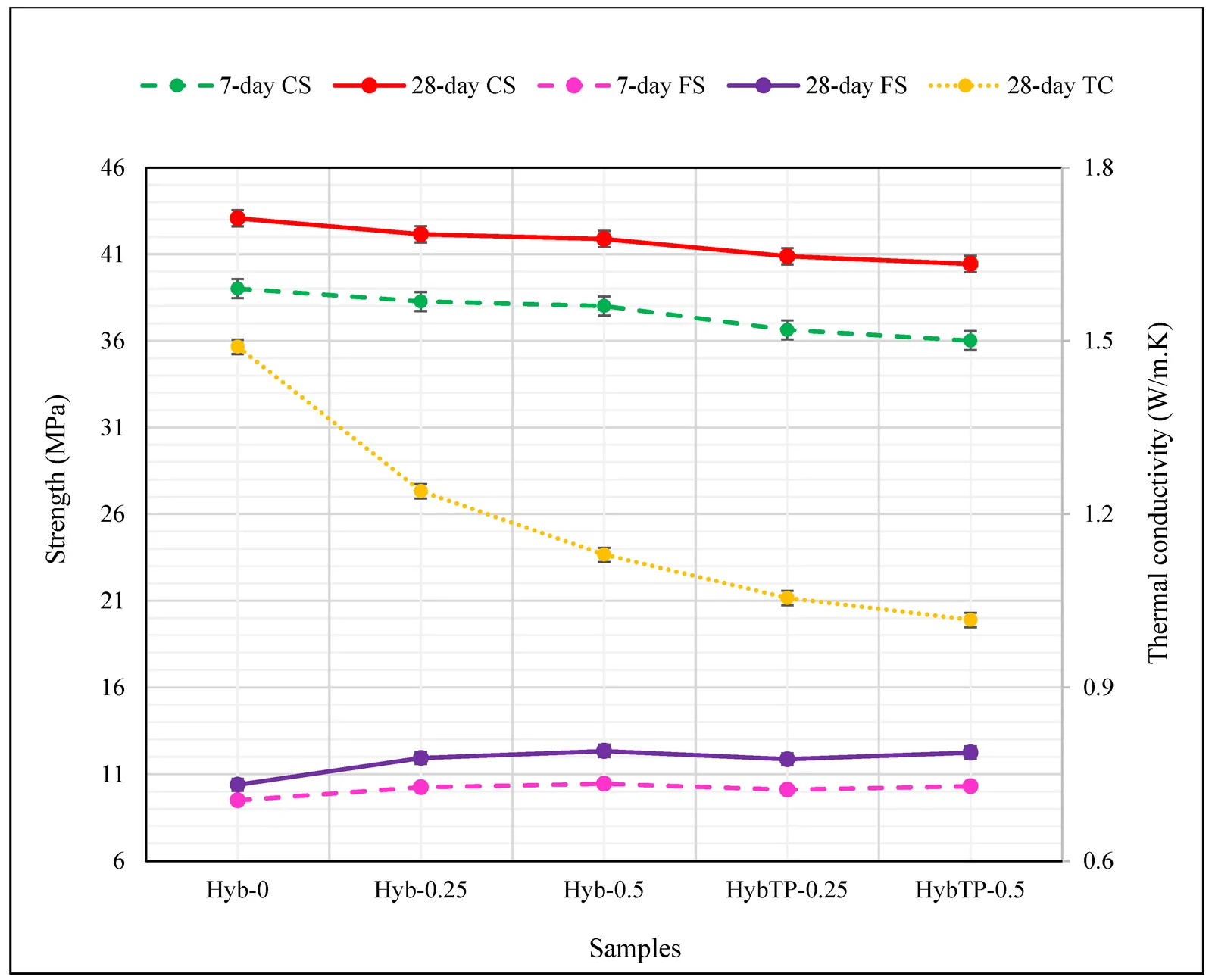
Compressive-flexural-thermal conductivity of mortars.

Figure 2 illustrates the comparative results of the compressive strength (CS) and flexural strength (FS) of mortar samples incorporating KCSFs and WTRP after 7 and 28 days, alongside the thermal conductivity (TC) measured after 28 days of curing, with error bars representing the standard deviations of replicate measurements. Following evaluation of the 7- and 28-day curing results, a tendency towards a decrease in compressive strength was observed in all hybrid mortar mixes compared to the reference mixture. In mixes containing only seed fiber (Hyb series), an increase in fiber content led to a reduction in compressive strength relative to the reference sample: 1.92% and 2.58% for the 7-day results, and 2.16% and 2.78% for the 28-day results, respectively. However, considering the experimental variability, these relatively small differences should be interpreted as comparable compressive strength performance rather than as distinct changes in mechanical behavior. In hybrid mortar mixes including WTRP (HybTP series), the reduction in compressive strength is more noticeable, exhibiting declines of 6.12% and 7.71% after 7 days of curing, and 5.11% and 6.12% after 28 days of curing relative to the reference sample. Furthermore, the evaluation of seed fiber mixes revealed that the incorporation of waste tire powder diminished compressive strength. When samples with same seed fiber ratios were evaluated, mixes incorporating waste tire powder (HybTP series) exhibited reduced compressive strength relative to those containing solely seed fiber (Hyb series). The comparison of Hyb-0.25 and HybTP-0.25 samples indicated that the incorporation of WTRP resulted in a 4.28% reduction in compressive strength after 7 days of curing and a 3.01% reduction after 28 days of curing. When these results are evaluated together, increasing seed fiber content and incorporating WTRP were generally associated with a tendency toward reduced compressive strength. The water absorption capacity of natural fibers and adhesion issues at the fiber–matrix interface might restrict the continuity of the cement matrix and diminish load transmission efficiency. The heightened heterogeneity and facilitation of void formation in the composite structure due to increasing fiber content are primary factors explaining the reduction in compressive strength [39,43,48,49,50,51]. Because the nominal water-to-cement ratio was kept constant without compensating for fiber water uptake, the hydrophilic character of the natural fibers may have altered the effective water availability within the fresh matrix. Conversely, the hydrophobic nature of WTRP may have affected particle wetting, workability, and compaction rather than directly increasing water demand. These effects may influence hydration conditions, entrapped air, and matrix consolidation and, consequently, may contribute to the observed variations in compressive strength. Therefore, the mechanical response should be interpreted as the combined outcome of constituent characteristics and their influence on fresh-state behavior and effective water availability, rather than being attributed solely to intrinsic fiber– or rubber–matrix interactions. Similarly, the hydrophobic surface structure and low elastic modulus of WTRP can lead to weak bonding with the cement matrix, weakening the matrix-interface transition zone (ITZ). This situation results in stress concentrations when subjected to load, leading to a reduction in compressive strength. The results of this investigation align with existing literature, indicating that the enhanced strength loss in hybrid mortar mixtures with waste tire powder may be attributed to inadequate matrix–particle interaction and an increased void ratio [33,34,35,39,52]. It should also be considered that WTRP was introduced as a mass-based rather than a volume-based replacement of sand. Owing to the lower density of WTRP relative to natural sand, this substitution increases the volumetric contribution of the rubber phase and may consequently influence the bulk density, compaction, and pore structure of the mortar. These mixture-design effects may contribute to the observed changes in mechanical and thermal performance and should therefore be considered together with the intrinsic characteristics of WTRP when interpreting the results.

The literature indicates that natural fiber reinforcing in cement-based composites enhances flexural strength by restricting microcrack development. Moreover, the regenerative characteristics and low price of natural fibers render them interesting as sustainable and economical alternatives [53]. An examination of the flexural strength results revealed a notable increasing trend in all hybrid mortar mixes relative to the reference mixture. In comparison to the reference sample, the 7-day outcomes exhibited increases of 7.89% and 10%, while the 28-day results demonstrated increases of 14.8% and 18.65%. This result clearly demonstrates the positive effect of increasing seed fiber content on flexural performance. In hybrid mortar mixtures containing WTRP (HybTP series), an increase in flexural strength was observed compared to the reference sample. This increase was determined to be 6.42% and 8.52%, respectively, after 7 days of curing, and 14.13% and 17.79% after 28 days of curing. Nonetheless, while comparing mixes with same fiber content ratios, it was determined that the samples incorporating WTRP exhibited a lower increase compared to those having only seed fiber. In the comparison of Hyb-0.25 and HybTP-0.25, the incorporation of WTRP reduced the flexural strength by 1.36% after 7 days of curing and by 0.58% after 28 days of curing. Similarly, in the comparison of Hyb-0.5 and HybTP-0.5, the differences were found to be 1.33% and 0.72%, respectively. The comparatively modest reduction in flexural strength following WTRP incorporation differs from the more pronounced reductions reported in previous studies [28,38,39]. This difference may be associated with variations in rubber content, particle characteristics, and mixture design. In the present study, WTRP was incorporated together with KCSFs, whose crack-bridging contribution may have mitigated the adverse influence of the rubber–matrix interface on flexural performance. However, considering the relatively small differences between the corresponding Hyb and HybTP mixtures, the contribution of WTRP to the observed changes in flexural strength cannot be conclusively distinguished from experimental variability. This suggests that natural fibers enhance the flexural strength of the cement-based matrix; however, the incorporation of WTRP partially diminishes this positive impact. This finding aligns with the literature indicating that natural fibers enhance flexural performance via crack-bridging processes and fiber tensile energy [38,54,55,56]. The capacity of fibers to impede microcrack propagation under stress and enhance energy absorption is the basic mechanism for the increase of flexural strength. However, the literature indicates that the addition of waste tire dust can lead to weak bonding and local discontinuities in the matrix–interface transition zone (ITZ), which partially limits the load transfer efficiency [37,38]. The findings of the current study align with this tendency, indicating that KCSFs significantly enhance flexural strength, whereas WTRP limits this enhancement. Sarıkaya et al. [43] examined the influence of kapok fiber on cement-based mortars and reported an increase in flexural strength of up to 9.50% and a reduction in thermal conductivity of approximately 19% with the incorporation of 1% kapok fiber. Similarly, Susurluk et al. [57] observed enhancements in flexural strength between 5.91% and 15.76%, associated with increased fiber content in ground granulated blast furnace slag and fly ash mortars incorporating kapok fibers, while indicating that declines in compressive strength were between 3.65% and 14.82%. This research indicates that the 28-day flexural strength improved by 14.80–18.65% in mixtures solely comprising kapok–cotton fibers, whereas the decline in compressive strength was confined to 2.16–2.78%, indicating improved flexural strength accompanied by relatively small reductions in compressive strength.

The thermal conductivity values measured after 28 days of curing were determined to be 1.49, 1.24, 1.13, 1.055 and 1.017 W/mK for the Hyb-0, Hyb-0.25, Hyb-0.5, HybTP-0.25 and HybTP-0.5 series, respectively. When the thermal conductivity results were evaluated, it was concluded that the thermal conductivity values diminished over all hybrid mortar series in comparison to the reference mixture. In mixes involving only KCSFs (Hyb-0.25 and Hyb-0.5), a decrease of 16.77% and 24.16%, respectively, was observed after 28 days of curing in comparison to the reference sample (Hyb-0). This result clearly demonstrates the effect of increasing fiber content on improving insulation performance. In hybrid mortar mixtures with WTRP (HybTP-0.25 and HybTP-0.5), the reduction in thermal conductivity was significant, with decreases of 29.19% and 31.74%, respectively, noted in the 28-day results. In comparisons of mixes with same seed fiber ratios, it was determined that samples with WTRP had superior insulating performance relative to those composed only of KCSFs. A comparison of Hyb-0.25 and HybTP-0.25 revealed an additional insulation enhancement of approximately 14.91%, while a comparison of Hyb-0.5 and HybTP-0.5 indicated an additional insulation enhancement of approximately 10%. In this study, the lowest thermal conductivity was obtained with the HybTP-0.5 sample, corresponding to a thermal conductivity reduction of 31.74% compared to the reference sample. Nevertheless, despite the 31.74% reduction in thermal conductivity, a decline in flexural strength was also observed. In this case, optimizing enhancing mechanical strength and thermal insulation will be basic for the use of the hybrid mortar designs discussed in this study in applications in engineering. The results indicate that the incorporation of seed fiber with WTRP provides enhanced insulation performance relative to systems utilizing only seed fiber. This trend aligns with literature indicating that rubber-based additives might diminish thermal conductivity due to their low-density structure and the disruption of the continuous phase responsible for the transfer of heat, which creates micro-discontinuities within the matrix [27,28,29,37,52,58]. Similarly, it has been widely reported in the literature that natural fibers enhance the insulating properties of cement-based composites owing to their low densities, hollow structures and low thermal conductivity [16,17,43,59]. Sarıkaya et al. [43] reported an improvement of up to approximately 19% in the thermal insulation performance of cement-based mortars containing 1% kapok fiber. Similarly, Susurluk et al. [57] determined that as the kapok fiber content increased from 0.5% to 1%, thermal conductivity decreased by 5.72–10.67% in ground granulated blast furnace slag-based mortars and by 9.37–19.64% in fly ash-based mortars. In this study, the reduction in thermal conductivity by 16.77–24.16% in mixtures utilizing only kapok–cotton fibers is consistent with literature findings regarding the positive impact of natural fibers on thermal performance. The fact that this reduction reaches the level of 29.19–31.74% when WTRP is used in conjunction with fibers indicates that the combined use of fiber and rubber-based components can exert a more pronounced effect in limiting heat transfer. These findings indicate that the seed fiber–WTRP system could constitute a viable approach for multi-component mortar designs aimed at enhancing thermal insulation properties without completely compromising mechanical performance.

### 3.2. Scanning Electron Microscope (SEM) Analysis

**Figure 3:**
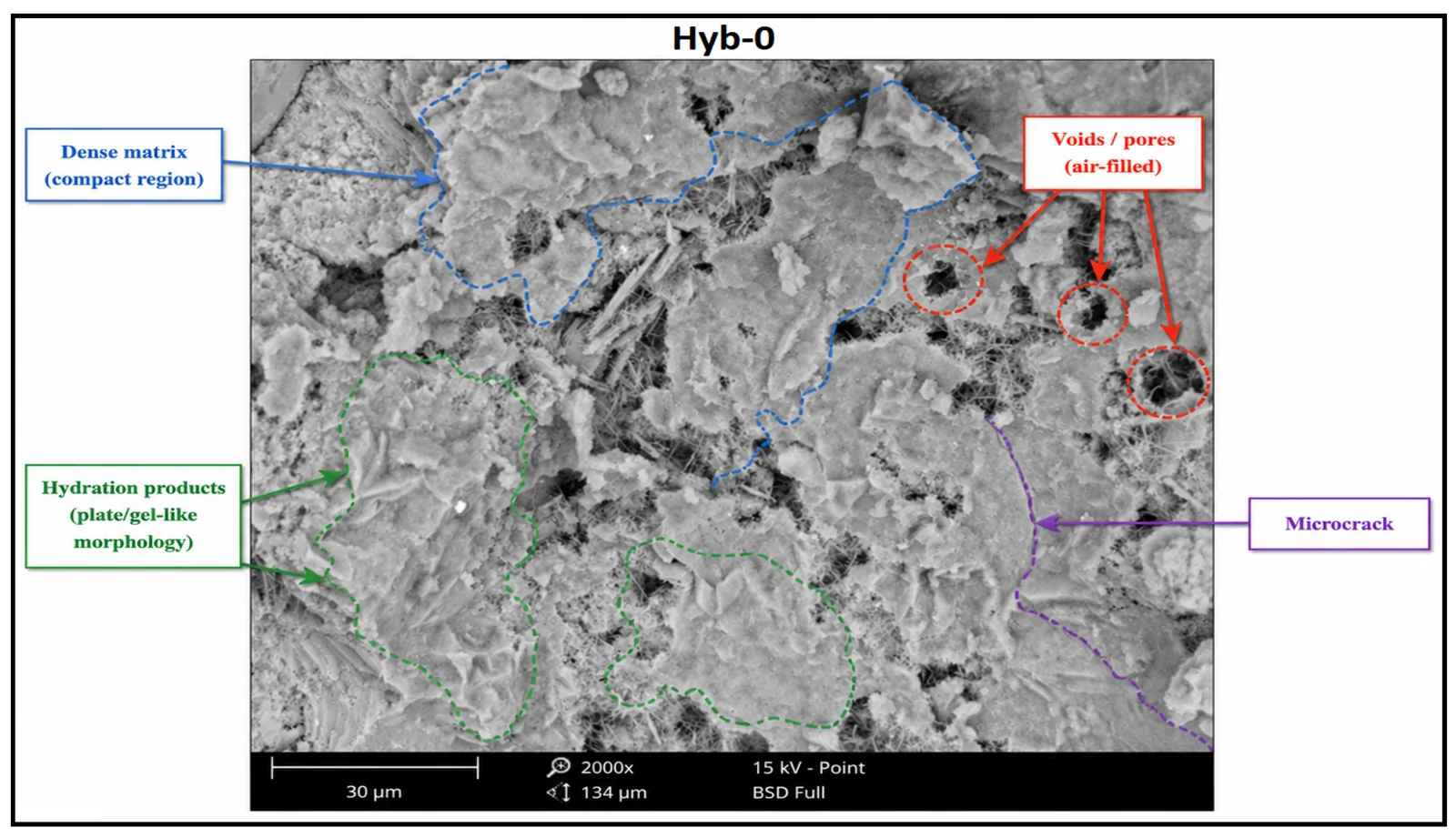
SEM micrograph depicting the microstructural morphology of Hyb-0 mortar sample.

**Figure 4:**
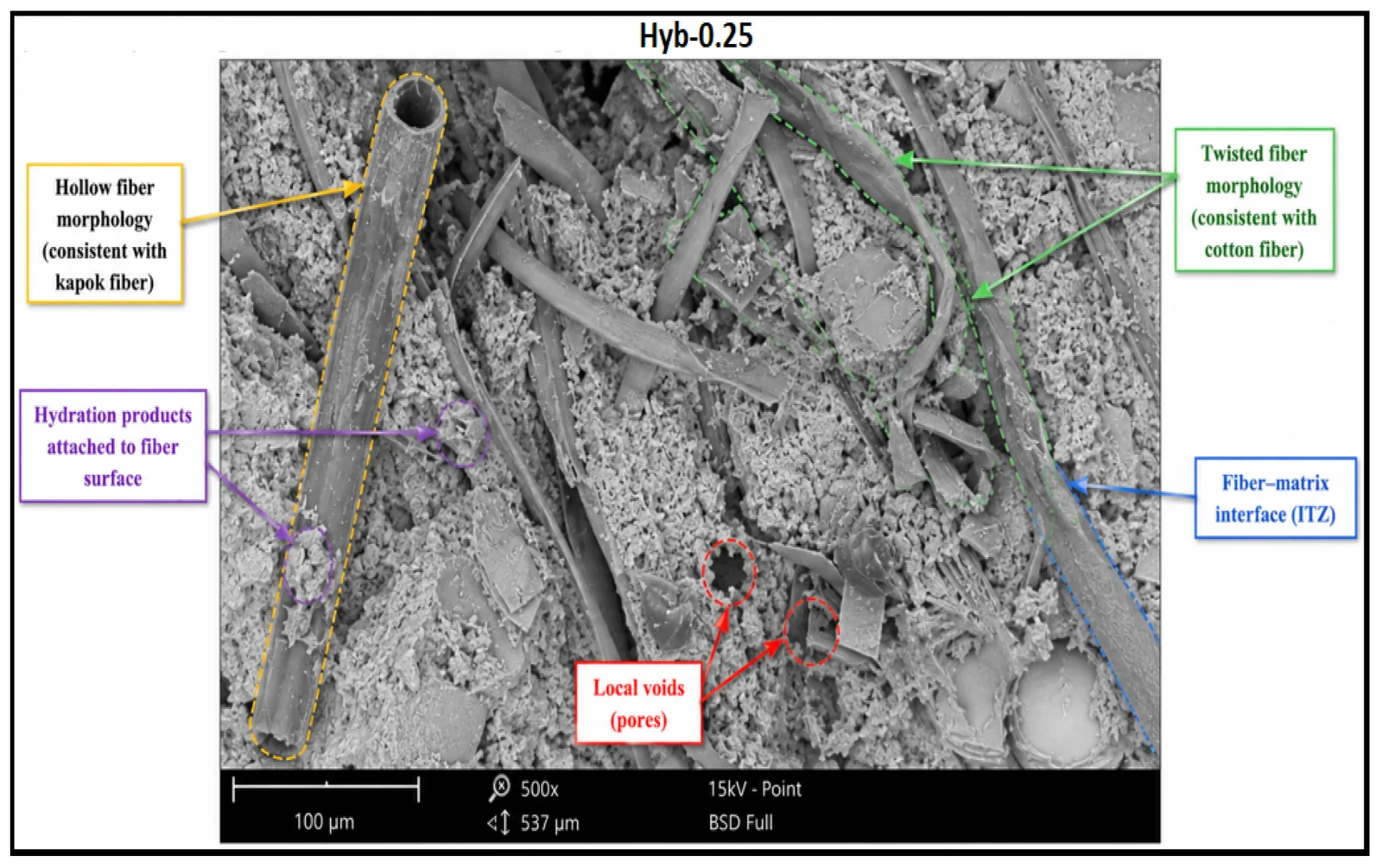
SEM micrograph depicting the microstructural morphology of Hyb-0.25 mortar sample.

**Figure 5:**
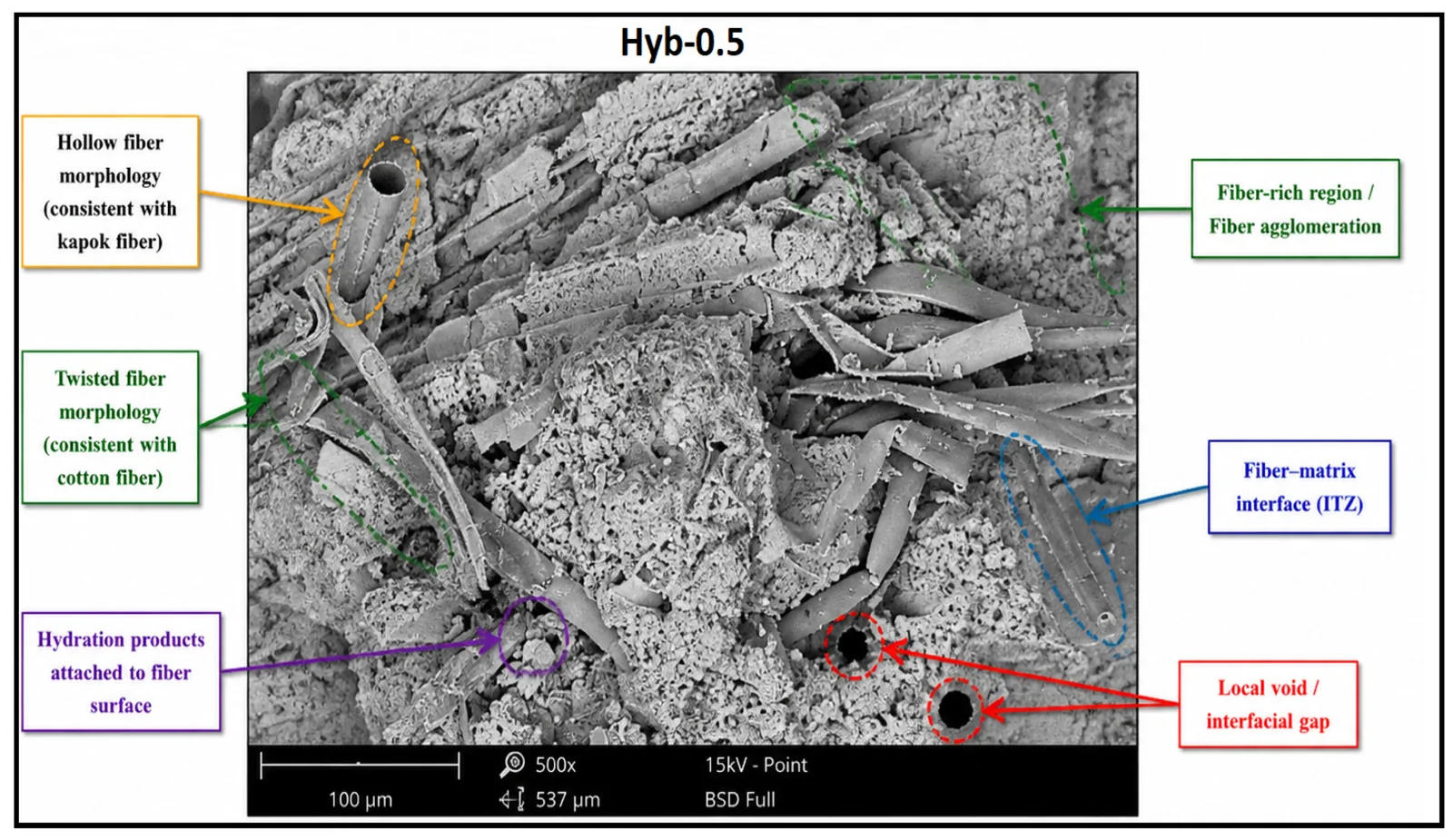
SEM micrograph depicting the microstructural morphology of Hyb-0.5 mortar sample.

**Figure 6:**
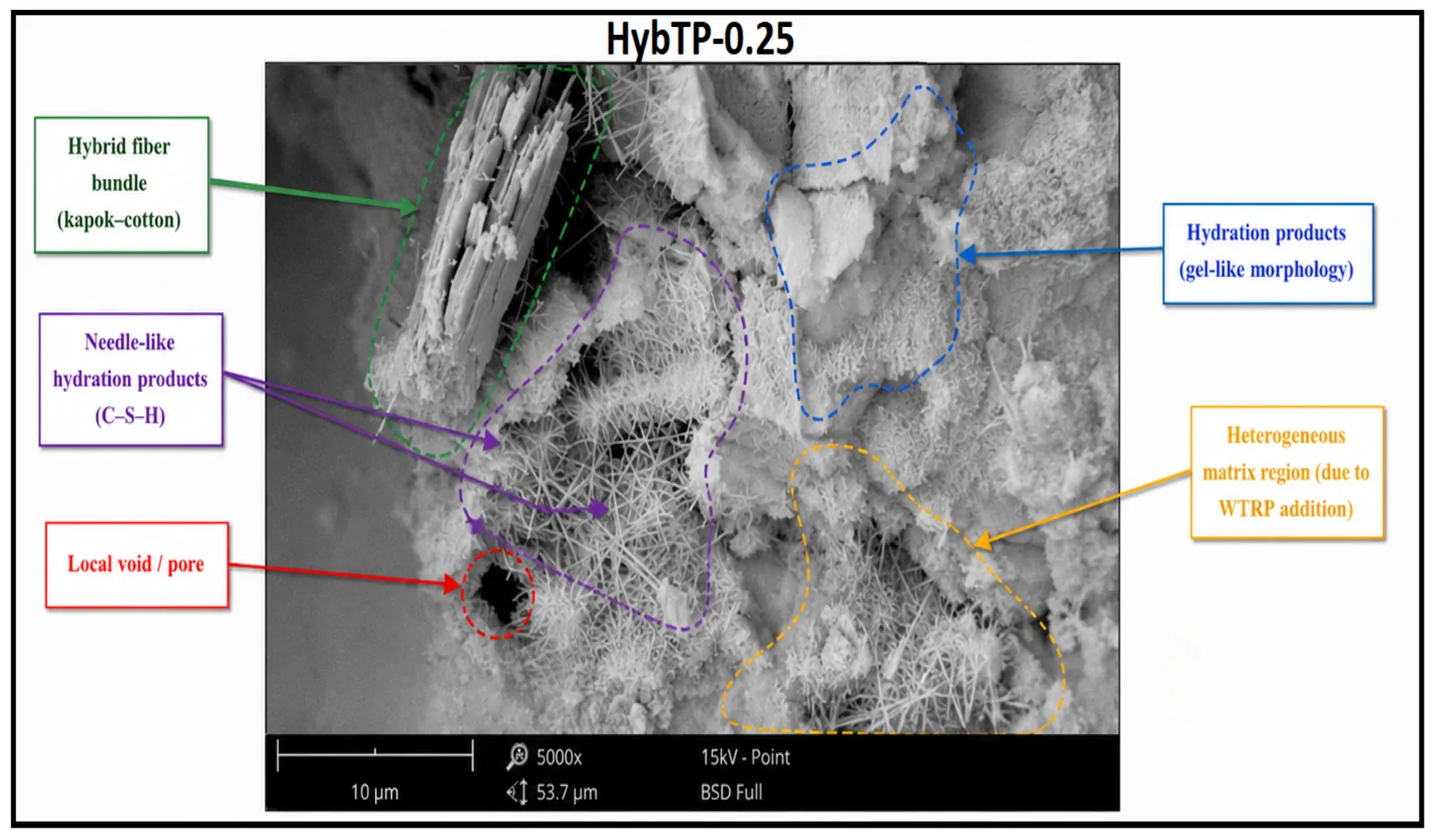
SEM micrograph depicting the microstructural morphology of mortar sample.

**Figure 7:**
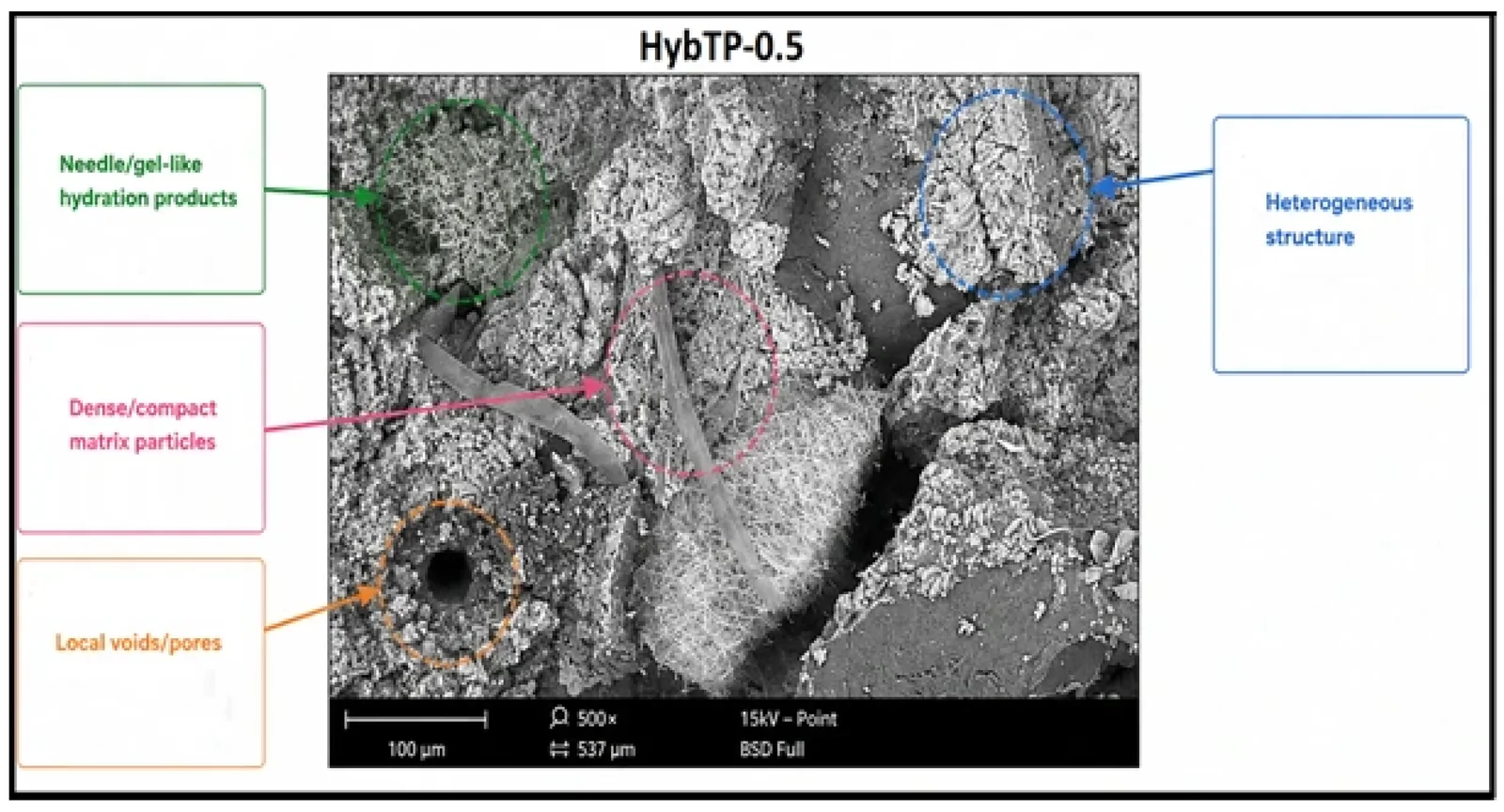
SEM micrograph depicting the microstructural morphology of HybTP-0.5 mortar sample.

Figure 3, Figure 4, Figure 5, Figure 6 and Figure 7 display SEM images of five different mortar mixtures. SEM analysis was utilized to evaluate the comparative impacts of KCSFs and WTRP on the microstructural characteristics of mortars. In this context, the compactness and continuity of the matrix, pore and microcrack formation, morphology and distribution of hydration products, and fiber–matrix and WTRP–matrix interface (ITZ) regions were investigated. Furthermore, microstructural changes were evaluated in samples incorporating KCSFs, considering the arrangement of fibers inside the matrix, the hydration products developing around the fibers, and fibers and matrix adhesion.

Figure 3 displays a SEM micrograph illustrating the microstructural morphology of the Hyb-0 reference sample. In the reference sample, which does not contain fiber or waste tire powder, it is observed that the hydration products are widely distributed within the matrix, with dense and compact regions forming in places. However, the microstructure was not entirely homogeneous, and localized pores, micro-scale discontinuities, and limited microcrack formation were also observed. The gel/plate-like and needle-like morphologies observed in some areas indicate the presence of hydration products resulting from cement hydration. Overall, the Hyb-0 sample exhibits a relatively coherent cement-based matrix structure dominated by hydration products, lacking a distinct fiber–matrix or rubber–matrix interface. This reference microstructure provides basis for the comparative assessment of morphological changes generated by the incorporation of KCSFs and WTRP.

Figure 4 exhibits a SEM micrograph demonstrating the microstructural morphology of the Hyb-0.25 sample. The incorporation of KCSFs resulted in a more heterogeneous microstructure compared to the Hyb-0 reference sample, with the fibers distributed in different orientations within the cement-based matrix. The SEM image reveals longitudinally extended fiber structures displaying a lumen/void morphology, indicative of the characteristic hollow structure of kapok fiber, whereas the more irregular and twisted forms align with the structural characteristics of cotton fiber [19,21]. It is observed that some regions of the fiber surfaces are coated with hydration products and fiber–matrix contact is developed, while local voids and interfacial discontinuities are also present around the fibers. The hydrophilic character of natural fibers and the interactions at the fiber–matrix interface can also be effective in the formation of such local voids and in matrix continuity, as reported in the literature [49,50,51,60]. Nonetheless, the fact that the fibers extend in different directions within the matrix and form a network that can interrupt crack propagation paths is consistent with studies reporting that natural fibers enhance flexural performance via a crack-bridging mechanism [39,61]. In general, these microstructural characteristics demonstrate behavior consistent with the limited compressive strength loss and accompanying flexural strength increase observed in the Hyb-0.25 sample compared to Hyb-0.

Figure 5 depicts a SEM micrograph exhibiting the microstructural morphology of the Hyb-0.5 sample. In comparison to the Hyb-0.25 sample, it is noted that the fiber density inside the matrix increases significantly with increasing seed fiber content, and in some areas, the fibers are positioned close together, forming local fiber concentrations. Distinct morphologies characteristic of kapok and cotton fibers were observed inside the matrix, revealing that certain areas of the fiber surfaces were covered with hydration products and that the interaction between the fibers and the matrix remained. Nevertheless, with increasing fiber content, the local voids surrounding the fibers and the bonding irregularities at the fiber–matrix interface appear to become pronounced in comparison to the Hyb-0.25 sample. This aligns with the literature indicating that elevated natural fiber content may prevent uniform distribution within the matrix, resulting in fiber clustering, localized voids and the formation of bonding discontinuities at the fiber–matrix interface [49,51]. Furthermore, the distribution of fibers in different directions within the matrix created a more extensive fiber network that could limit crack propagation, aligning with research indicating that natural fibers improve flexural performance through a crack-bridging mechanism [39,61]. Therefore, the microstructure of the Hyb-0.5 sample suggests that the more extensive fiber network formed by increasing seed fiber content supports the increase in flexural strength; conversely, the local voids and bonding discontinuities observed at the fiber–matrix interface may be associated with the limited decrease in compressive strength.

Figure 6 demonstrates a SEM micrograph displaying the microstructural morphology of the HybTP-0.25 sample. Compared to the Hyb-0.25 sample, which has the same seed fiber content but does not contain WTRP, the HybTP-0.25 sample exhibits a more heterogeneous matrix structure, with more pronounced local voids and microstructural discontinuities. However, it was observed that needle-like and gel-like hydration products remained within the matrix, but their distribution was irregular in places. This morphological change is consistent with the literature reporting that the hydrophobic character of WTRP, its low rigidity, and limited interface interaction with the cement matrix can weaken ITZ continuity and promote local void formation [1,27,28]. Conversely, the retention of the different orientations of the KCSFs within the matrix indicates that the fiber bridging mechanism, which can limit crack propagation, persists even in the presence of WTRP. Therefore, although the incorporation of WTRP partially restricted matrix homogeneity and interfacial continuity, the reinforcement mechanism provided by the KCSFs contributed to the preservation of flexural performance. Furthermore, the observed local voids and microstructural discontinuities, along with the low thermal conductivity of WTRP, are consistent with the lower thermal conductivity value obtained compared to Hyb-0.25, and similar thermal behavior has been reported in cement-based systems incluiding WTRP [28,29].

Figure 7 presents a SEM micrograph showing the microstructural morphology of the HybTP-0.5 sample. In comparison to the HybTP-0.25 sample, it is observed that microstructural heterogeneity becomes more pronounced with increasing seed fiber content, and matrix continuity is interrupted in places. The SEM image reveals compact matrix regions as well as irregular surface morphologies, localized voids and hydration products of varying densities. This appearance is consistent with the more heterogeneous and porous microstructural properties reported in cementitious systems incorporating [1,27]. Moreover, the enhanced flexural strength maintained despite the increased fiber ratio demonstrates that KCSFs continue providing a restraining influence on crack propagation [39,61]. Conversely, localized disruptions in matrix continuity may correlate with a limited reduction in compressive strength. The lowest thermal conductivity observed in the HybTP-0.5 sample can be attributed to the more heterogeneous and porous microstructure formed with WTRP and a higher fiber content, which inhibits heat-transfer mechanism within the solid phase [1,28,29].

Overall, SEM analyses have revealed that the compact and continuous matrix structure in the Hyb-0 reference sample is modified into a more heterogeneous morphology with the inclusion of seed fiber and WTRP. While the distribution of KCSFs within the matrix and their crack propagation-limiting effect were consistent with improved flexural performance, the localized voids and microstructural discontinuities that became apparent in WTRP-containing samples were associated with a limited decrease in compressive strength. These microstructural changes are consistent with previous SEM observations reporting that natural fibers can contribute to the crack-bridging mechanism within the cement-based matrix, whereas rubber particles due to their hydrophobic nature and limited interfacial adhesion with the cement matrix can create weaker ITZ regions and localized voids [37,39,43]. In contrast, the increased heterogeneity and localized porosity associated with the incorporation of fibers and WTRP may contribute to reduced continuity of heat-transfer paths, which is consistent with the experimentally observed reduction in thermal conductivity. Consequently, the SEM observations provide qualitative microstructural evidence that is consistent with the observed changes in mechanical and thermal performance, particularly with respect to matrix continuity, fiber–matrix interaction, and WTRP–matrix interfacial characteristics.

The SEM observations in the present study were used for qualitative assessment of matrix morphology, localized void formation, and constituent–matrix interfacial characteristics. As quantitative image analysis and EDS mapping were not included in the experimental program, parameters such as fiber spacing and orientation, pore area fraction, and interfacial transition zone dimensions were not quantitatively resolved. Accordingly, the observed microstructural features are interpreted as complementary evidence rather than as quantitative predictors of mechanical performance. Establishing quantitative relationships between pore structure and macroscopic properties would require additional multiscale characterization, including quantitative image analysis and complementary techniques capable of resolving pore populations across different length scales, and is therefore considered an important direction for future investigation.

### 3.3. Surface Area Analysis (BET)

BET analyses were performed to characterize the changes in the pore structure of mortar samples containing kapok–cotton fibers and WTRP. Within the scope of the analysis, the specific surface area, total pore volume and average pore diameter values of the samples were determined. The impact of increased seed fiber content on pore structure was assessed through Hyb-0, Hyb-0.25, and Hyb-0.5 samples, while the changes observed in the pore characteristics of the fiber-containing matrix due to 10% WTRP replacement were analyzed utilizing HybTP-0.25 and HybTP-0.5 samples. BET results were used to evaluate the relationship between quantitative pore parameters and the fiber–matrix and WTRP–matrix interface characteristics, void formation, and matrix heterogeneity observed in SEM analysis.

**Figure 8:**
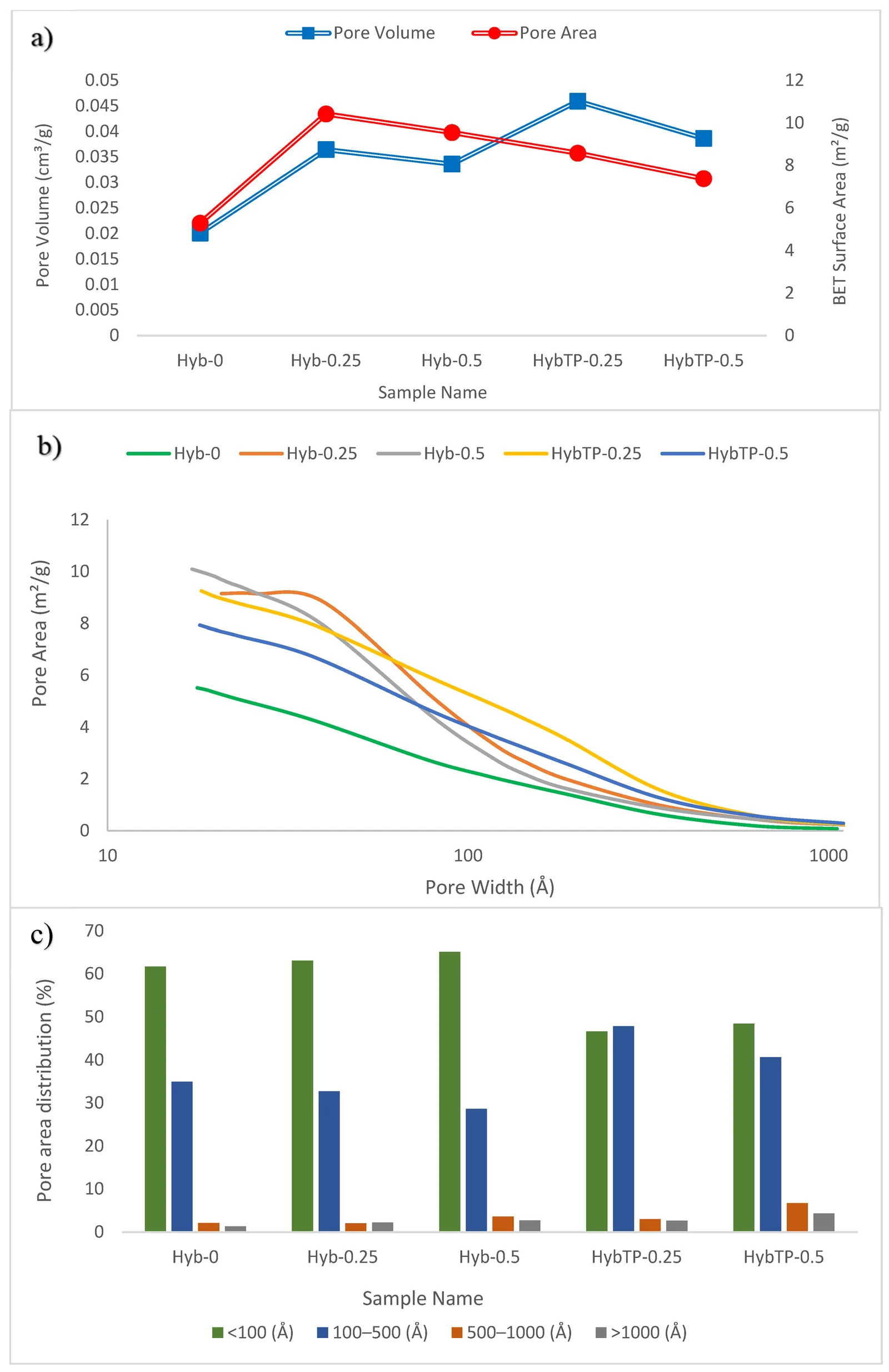
BET analysis of mortar samples containing KCSFs and WTRP. (**a**) BET surface area and total pore volume values of mortar samples containing KCSFs and WTRP. (**b**) Pore-size distribution curves. (**c**) Pore area distribution as a function of pore width.

Figure 8a illustrates the BET specific surface area and total pore volume of the samples; Figure 8b depicts the distributions of pore sizes; and Figure 8c reveals the contributions of different pore diameter ranges to the total pore area. Analyses reveal that the incorporation of kapok–cotton fibers and WTRP significantly modifies the pore structure of the mortars. Compared with Hyb-0, higher BET specific surface area and total pore volume values were observed for the Hyb-0.25 and Hyb-0.5 samples, with the largest increase in specific surface area observed for Hyb-0.25. However, because BET analysis was performed on a single specimen from each mixture, these differences are considered descriptive observations and their reproducibility cannot be statistically established. However, the fact that fine pores with diameters of <100 Å remain dominant in fiber-containing samples indicates that the addition of KCSFs alters not only the total pore volume but also the internal surface area and pore-size distribution. It has been reported in previous studies that natural fibers can change the pore structure in cement-based systems due to their water absorption characteristics, surface morphologies, and fiber–matrix interfaces [62]. Therefore, the observed pore characteristics may also be influenced by variations in effective water availability, air entrainment, and compaction associated with fiber incorporation, in addition to the intrinsic characteristics of the fibers. However, although the BET specific surface area and total pore volume remained above those of the Hyb-0 reference in both mixtures containing KCSFs, no linear or unidirectional change was observed in these parameters when the fiber content was increased from 0.25 to 0.5. This suggests that the enhancement of seed fiber content does not merely modify the pore structure through a straightforward the amount effect by increasing or decreasing pore quantity but is rather influenced by fiber distribution, the development of fiber–matrix interfaces, and variations in the relative contributions of pores of different sizes. Therefore, the influence of the fiber incorporation ratio on the pore structure must be assessed alongside the pore-size distribution, rather than examining total pore volume or specific surface area in isolation. In instances of HybTP samples with WTRP, it is significant that the surface area and total pore volume exhibit distinct trends. When comparing Hyb and HybTP mixtures with the same seed fiber ratio, it was determined that the incorporation of WTRP resulted in an increase in total pore volume, despite a decrease in BET surface area. The fact that these two parameters change in opposite directions indicates that the effect of WTRP on the pore structure cannot be explained solely by the change in the total pore volume; changes in the pore-size distribution must also be taken into account. While small pores contribute to high specific surface area, larger pores can provide a greater volumetric contribution; thus, the redistribution of pore area toward wider diameter ranges in mixtures containing WTRP may explain the simultaneous observation of a lower BET specific surface area and a higher total pore volume. The HybTP-0.25 sample demonstrates a higher total pore volume, but the HybTP-0.5 sample shows a slight decrease in pore volume, though its distribution extends a larger diameter range. Specifically for HybTP-0.5, the surface area contribution from pores under 100 Å diminished to around 48%, while the contribution from pores ranging from 100 to 500 Å increased to about 41%, and the total contribution from pores over 500 Å reached approximately 11%. This change indicates that the incorporation of WTRP significantly affects total pore volume and pore-size distribution, resulting in a wider range of pore sizes.

When BET results are assessed alongside with SEM observations, it becomes clear that KCSFs and WTRP effectively restructure the pore structure by generating new interfacial zones inside the matrix. The porous structure of natural fibers and the characteristics of the fiber–matrix interface can play a significant role in the microstructural development of cement-based composites; furthermore, recent studies have demonstrated that fiber properties can change hydration and pore-size distribution [63]. In this context, the changes in pore-size distribution determined by BET analysis and the fiber–matrix and WTRP–matrix interface characteristics observed in SEM images provide mutually supporting microstructural findings. These changes in the pore structure are also consistent with mechanical and thermal performance. Compared to Hyb-0, the 28-day compressive strength of the samples containing only KCSFs decreased by 2.16–2.79%, while flexural strength increased by 14.81–18.65% and thermal conductivity decreased by 16.78–24.16%. Although the reduction in compressive strength for the HybTP-0.25 and HybTP-0.5 samples containing WTRP reached approximately 5.11% and 6.13% respectively, the flexural strength remained above that of the reference sample, while the reduction in thermal conductivity reached approximately 29.19% and 31.74% respectively. While the crack-bridging mechanism of natural fibers contributes to the flexural behavior, the changed pore structure and interface regions resulting from the addition of fibers and WTRP can be associated with a limited loss in compressive strength. It is reported in the literature that natural fibers can enhance the flexural and fracture behavior of cement-based composites, whereas changes in the fiber–matrix interface and pore structure can affect compressive performance [64]. On the other hand, the rearrangement of the pore structure and the development of a larger pore-size distribution may have contributed to the lower thermal conductivity values observed in samples containing WTRP by limiting continuous heat-transfer paths within the solid matrix. In conclusion, a combined evaluation of BET, SEM, and mechanical and thermal test results indicates that kapok–cotton fibers enhance flexural performance while maintaining the predominance of fine pores within the BET-accessible pore structure. The incorporation of WTRP was associated with a shift toward a broader pore-size distribution, accompanied by a more pronounced reduction in thermal conductivity and a limited decrease in compressive strength.

It should be noted that N_2_ adsorption analysis primarily characterizes the accessible fine-pore structure within the analyzed material and does not capture the full pore hierarchy of mortar, particularly macroscopic voids and entrained air. Therefore, the BET-derived pore characteristics should not be regarded as a direct representation of the pore features governing the bulk thermal and mechanical behavior of the mortar. The observed thermal conductivity and flexural performance may additionally be influenced by macro-voids, air content, compaction, and the aggregate–paste skeleton, which were not quantified by the present BET analysis. Accordingly, the BET results are used here as complementary microstructural evidence rather than as a sole explanation for the measured macroscopic properties. Consequently, a direct quantitative relationship between the BET-derived pore parameters and thermal conductivity cannot be established from the present dataset.

Furthermore, because BET analysis was performed on the composite mortar specimens, the measured pore characteristics represent the combined contributions of the cementitious matrix, fibers, WTRP, and their associated interfacial regions; the individual contributions of these constituents cannot be resolved from the present measurements. Multiscale characterization using complementary techniques such as mercury intrusion porosimetry (MIP) and micro-computed tomography (micro-CT), together with separate characterization of the constituent materials, would enable a more comprehensive distinction of pore populations and their contributions. Consequently, the SEM findings support tmacroscopic performance. Such analyses are therefore recommended for future investigation.

### 3.4. X-Ray Diffraction (XRD) Analysis

**Figure 9:**
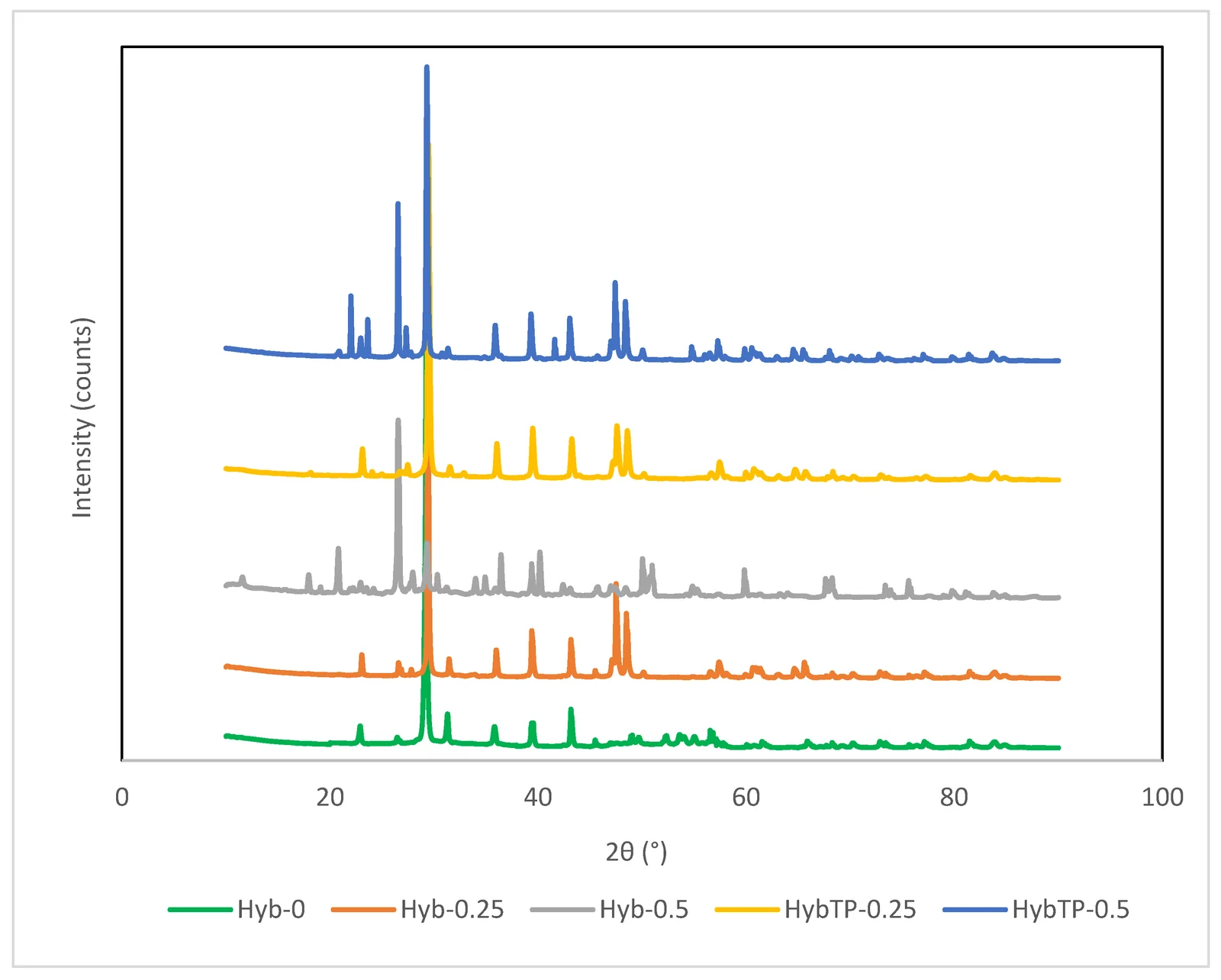
X-ray diffraction analysis of mortar samples.

XRD analysis was conducted to evaluate the impact of incorporating KCSFs and WTRP on the mineralogical structure of mortars and the formation of hydration products. The XRD patterns of Hyb-0, Hyb-0.25, Hyb-0.5, HybTP-0.25, and HybTP-0.5 samples are presented comparatively in Figure 9. Phase assessment considered not only peak intensities but also characteristic peak locations and broad diffraction regions associated with low-crystallinity hydration products. In cementitious systems, the prominent peak around 2θ = 26.6° is mainly related to the quartz (Q) phase, while the broad diffraction area observed in the 20–32° range is associated to low-crystallinity calcium silicate hydrate (C–S–H)-based binder products. The characteristic peak around 2θ ≈ 34–35° indicates the presence of the portlandite (CH) phase formed as a result of hydration [65]. While the effect of natural fibers on cement-based systems occurs mainly through fiber–matrix interface interaction and physical reinforcement mechanisms, the low reactivity and hydrophobic surface character of WTRP can limit the development of hydration products and the continuity of the binder matrix [27,66]. In this framework, XRD patterns were assessed comparatively, focusing on the identification of potential new crystalline phases as well as the retention of characteristic phases, peak regions, and relative changes in areas associated with hydration products.

The XRD pattern of the Hyb-0 reference sample reveals the characteristic mineralogical structure of the cement-based mortar system. The prominent peak observed in the pattern at approximately 2θ ≈ 26.6° has been associated with the quartz (Q) phase present in the mortar system. Nevertheless, the broad diffuse region observed in the 20–32° 2θ range has been attributed to the presence of a low-crystallinity C–S–H-based binder phase that developed as a result of cement hydration. The characteristic peak observed around 2θ ≈ 34–35° has been associated with the portlandite (CH) phase formed as a result of cement hydration. The retention of the characteristic peaks of the quartz (Q) phase signifies that this crystalline phase remains within the mineralogical structure despite hydration. In contrast, the observation of a diffuse region associated with a low-crystallinity C–S–H-based binder, along with CH peaks, reveals the development of characteristic hydration products of Portland cement in the Hyb-0 reference sample. It is stated that similar mineralogical properties have been reported in recent studies and that the low-crystallinity C–S–H based binder phase and the crystalline CH phase are among the characteristic phases of hydrated cement-based systems [67]. Accordingly, the phase distribution determined in the Hyb-0 sample was used as a reference in the comparative evaluation of the changes in the mineralogical structure resulting from the incorporation of seed fiber and WTRP.

The XRD pattern of the Hyb-0.25 sample indicates that the basic mineralogical phases detected in the Hyb-0 reference sample are largely preserved. The prominent peak at approximately 2θ ≈ 26.6° has been attributed to the quartz (Q) phase, the low crystalline region between 20 and 32° to the C–S–H-based binder phase, and the peak around 2θ ≈ 34–35° to the portlandite (CH) phase. The lack of new and distinct crystalline phase, even with the incorporation of KCSFs, suggests that the low fiber content does not modify the basic mineralogical structure of the cement matrix. Nonetheless, it has been reported in the literature that cellulosic fibers can influence hydration kinetics and the development of hydration products through their water retention and nucleation effects [68]. Therefore, it is assessed that the changes observed in the Hyb-0.25 sample are related more to the relative evolution of existing hydration products and changes in phase distribution than to the formation of a new crystalline phase.

The XRD pattern of Hyb-0.5 sample contains similar basic mineralogical phases to Hyb-0.25, but differs in terms of the distribution of low crystalline components. In the Hyb-0.25 sample, the quartz (Q) peak around 2θ ≈ 26.6° exhibits a sharp and dominant character, while in the Hyb-0.5 sample, the diffuse contribution is more pronounced, especially in the 2θ range of 20–30°. This variation can be attributed to the differentiation in the relative contribution of low-crystallinity C–S–H-based hydration products, which signal in the region in question, to the XRD pattern. Recent studies have also reported that the low crystalline structure of C–S–H is characterized by a broad diffuse distribution in XRD patterns [69]. However, the preservation of characteristic peaks for the quartz (Q) phase at approximately 2θ ≈ 26.6° and the portlandite (CH) phase around 34–35°, and the failure to identify a new crystalline phase, indicate that increasing the seed fiber ratio does not cause a significant transformation in the basic crystalline phase composition. This observation is consistent with kapok and cotton fibers functioning as fiber reinforcement within the cement-based system, rather than as reactive mineral components that form new crystalline hydration products. Therefore, the impact of increased fiber content on the XRD pattern can be assessed mainly by the relative influence of low-crystallinity hydration products including C–S–H and changes in the distribution of existing phases, rather than the formation of new crystalline phase.

In the HybTP-0.25 sample, the inclusion of WTRP in the mixture resulted in differences in the relative peak intensities in the XRD pattern compared to Hyb-0.25, although the basic mineralogical phases were preserved. The dominant peak around 2θ ≈ 26.6° corresponds to the quartz (Q) phase, while the low crystalline region in the 20–32° range is associated with C–S–H-based hydration products, and the characteristic peak around 2θ ≈ 34–35° is associated with portlandite (CH). The absence of additional identifiable crystalline peaks indicates that the basic crystalline phase assemblage remained broadly similar within the resolution of the present XRD analysis. However, the observed differences in relative peak intensities may reflect changes in the relative distribution or development of existing hydration products, which cannot be quantified from the present qualitative XRD data alone. Current studies reveal that the limited interfacial compatibility of waste tire rubber with cement paste can affect the distribution of hydration products and matrix integrity [70].

In the HybTP-0.5 sample, the simultaneous presence of WTRP and higher seed fiber content led to more significant changes in the XRD pattern relative to HybTP-0.25. While the quartz (Q) peak around 2θ ≈ 26.6° retains its dominant character, the variation in relative peak intensities and distributions, particularly in the 20–30° range, indicates a change in the mineralogical phase distribution of hydration products. Although the low crystalline phase associated with C–S–H in the 20–32° range and the portlandite (CH) peak near 2θ = 34–35° were maintained, no additional crystalline phase was detected. WTRP’s hydrophobic surface and limited interfacial compatibility with cement paste can negatively affect the development of hydration products and matrix continuity [70]. This mineralogical change, when evaluated together with the heterogeneous structure and local voids identified in SEM images, reveals a microstructural trend consistent with the loss of compressive strength observed in WTRP-containing samples.

When XRD results are analyzed together with mechanical, thermal and microstructural results, it becomes clearly demonstrated that the incorporation of seed fiber and WTRP significantly influences mortar performance while preserving the basic crystalline phases. In relation to the Hyb-0 sample, mixtures consisting only of KCSFs exhibited a 28-day compressive strength reduction of 2.16–2.79%, although flexural strength displayed an enhancement of 14.81–18.65% and thermal conductivity diminished by 16.78–24.16%. In mixtures containing WTRP, although the loss in compressive strength increased to 5.11–6.13%, an increase in flexural strength of 14.13–17.79% was maintained, and a decrease in thermal conductivity of 29.19–31.74% was achieved. Recent studies also report that mechanical losses in cement mortars containing WTRP are related to the hydrophobic surface of rubber and the weak rubber–cement interface [71]. Changes in the pore-size distribution determined by BET analysis also support this mechanism, alongside the fiber–matrix and WTRP–matrix interfaces observed via SEM. Consequently, changes in phase distribution observed through XRD, changes in pore-size distribution determined by BET analysis and the heterogeneous microstructure revealed by SEM overall demonstrate that KCSFs enhance flexural performance, whereas WTRP makes a significant contribution to thermal insulation performance in exchange for limited compressive strength loss. It should be noted that the XRD analysis in the present study was used for qualitative phase identification and comparative assessment of the diffraction patterns. Although variations in relative peak intensities and diffuse regions were observed among the mixtures, these differences cannot be directly interpreted as quantitative changes in the contents of individual hydration products. In particular, the absence of additional identifiable crystalline peaks indicates only that no new crystalline phases were detected within the resolution of the present XRD analysis and does not exclude possible changes in the relative amounts or development of existing hydration products. Accordingly, the XRD results are interpreted as qualitative mineralogical evidence rather than as a quantitative measure of hydration extent.

### 3.5. Exploratory Relationships Among Mechanical, Thermal, and Pore Characteristics

**Figure 10:**
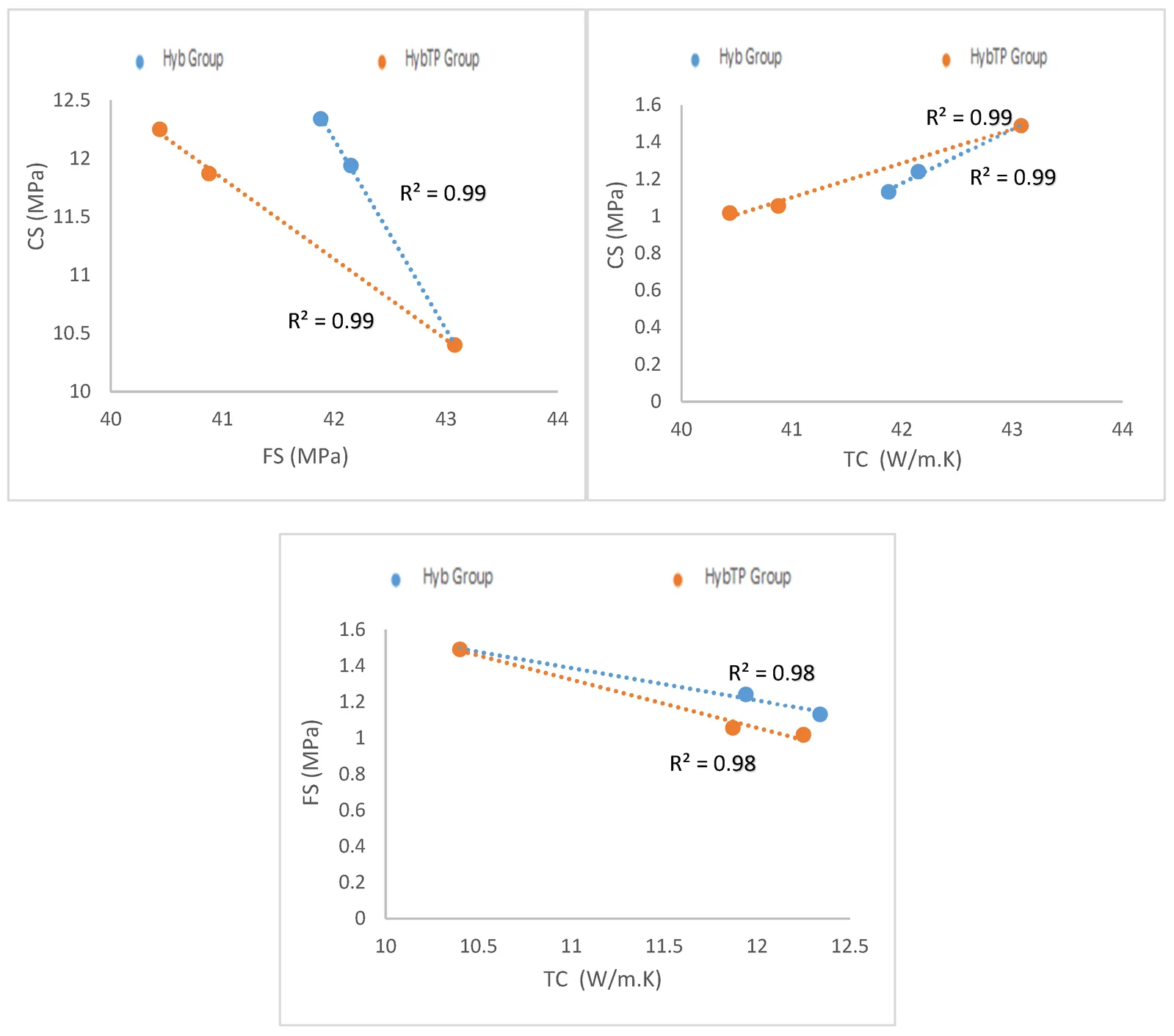
Correlations between the mechanical and thermal properties of Hyb and HybTP Groups.

In cementitious composites, the mechanical properties and thermal conductivity are closely related to the microstructural and porosity attributes of the material. Therefore, to comprehensively assess the impact of kapok–cotton fibers and WTRP on mortar performance, linear correlations among compressive strength (CS), flexural strength (FS), thermal conductivity (TC), and pore characteristics obtained from BET analysis were examined. Figure 10 depicts the correlations between the mechanical and thermal properties of the Hyb and HybTP groups, while Figure 12 and Figure 13 show the relationships between the pore characteristics and the mechanical and thermal performances of the Hyb and HybTP groups, respectively. Given the limited number of mixture-level observations within each group (n = 3), the regression analyses were used only as exploratory tools to visualize trends among the measured properties and were not interpreted as inferential statistical evidence.

Figure 10 illustrates that distinct linear correlations between mechanical and thermal parameters were obtained for both mixing groups. In the Hyb group, R^2^ values for the CS–FS, CS–TC, and FS–TC relationships were determined to be 0.99, 0.99 and 0.98, respectively, whereas in the HybTP group, these values were found to be 0.99, 0.99 and 0.98, respectively. These relationships demonstrate that changes in seed fiber and WTRP content simultaneously affect mechanical and thermal properties. The reduction in thermal conductivity which accompanies the improvement in flexural strength, particularly as the fiber content increases indicates that the crack-bridging effect of KCSFs, the interfacial zones formed by the fibers, and the changes in pore structure must be evaluated collectively. In the group containing WTRP, the fact that the reduction in thermal conductivity was accompanied by limited losses in compressive strength when evaluated alongside the pore and microstructural changes observed in BET and SEM analyses and the variations in phase distribution determined by XRD analysis demonstrates that the relationship between mechanical and thermal performance is consistent with the microstructural findings.

**Figure 11:**
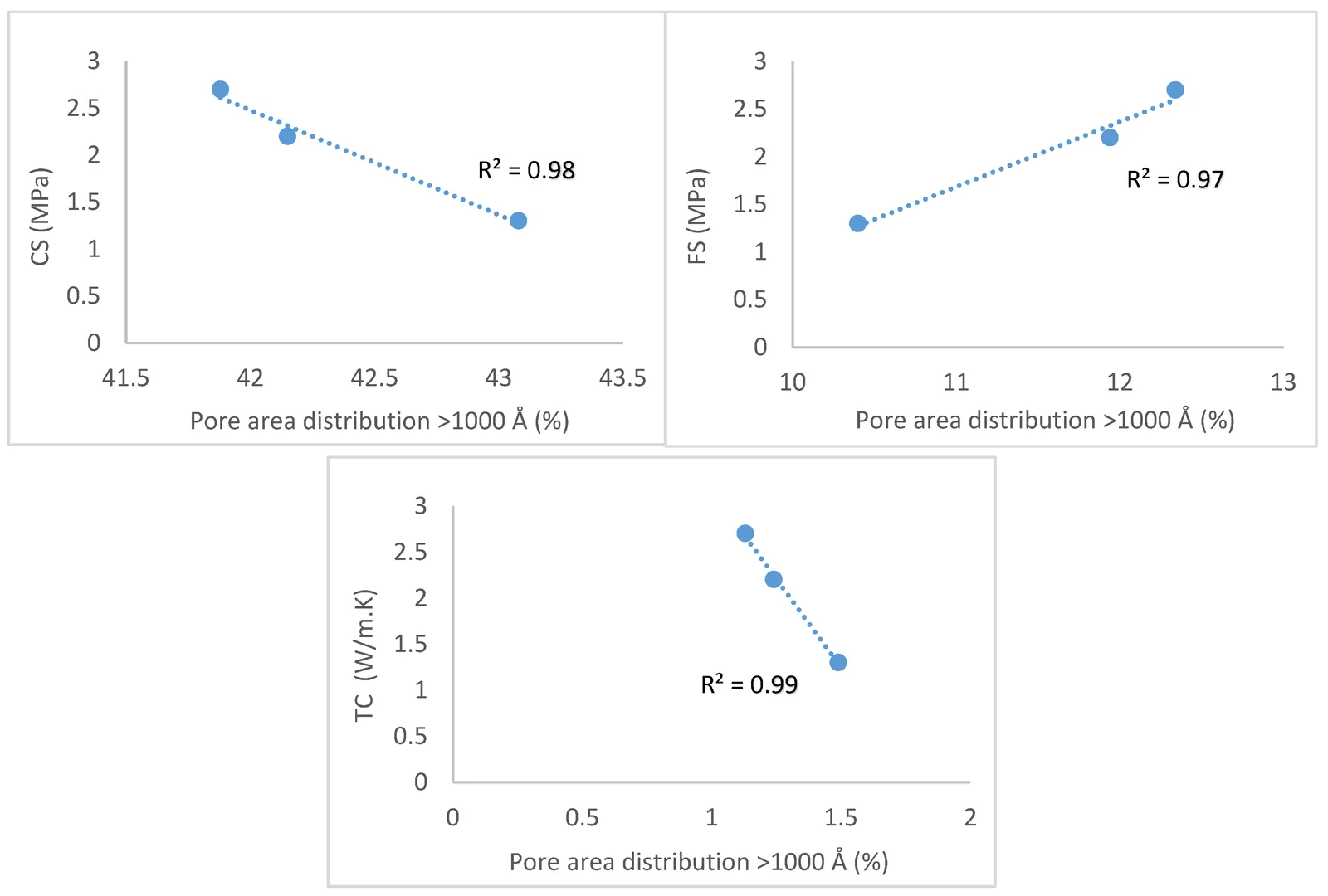
Correlations between the 1000 Å pore-size distribution and the mechanical and thermal properties of Hyb groups.

**Figure 12:**
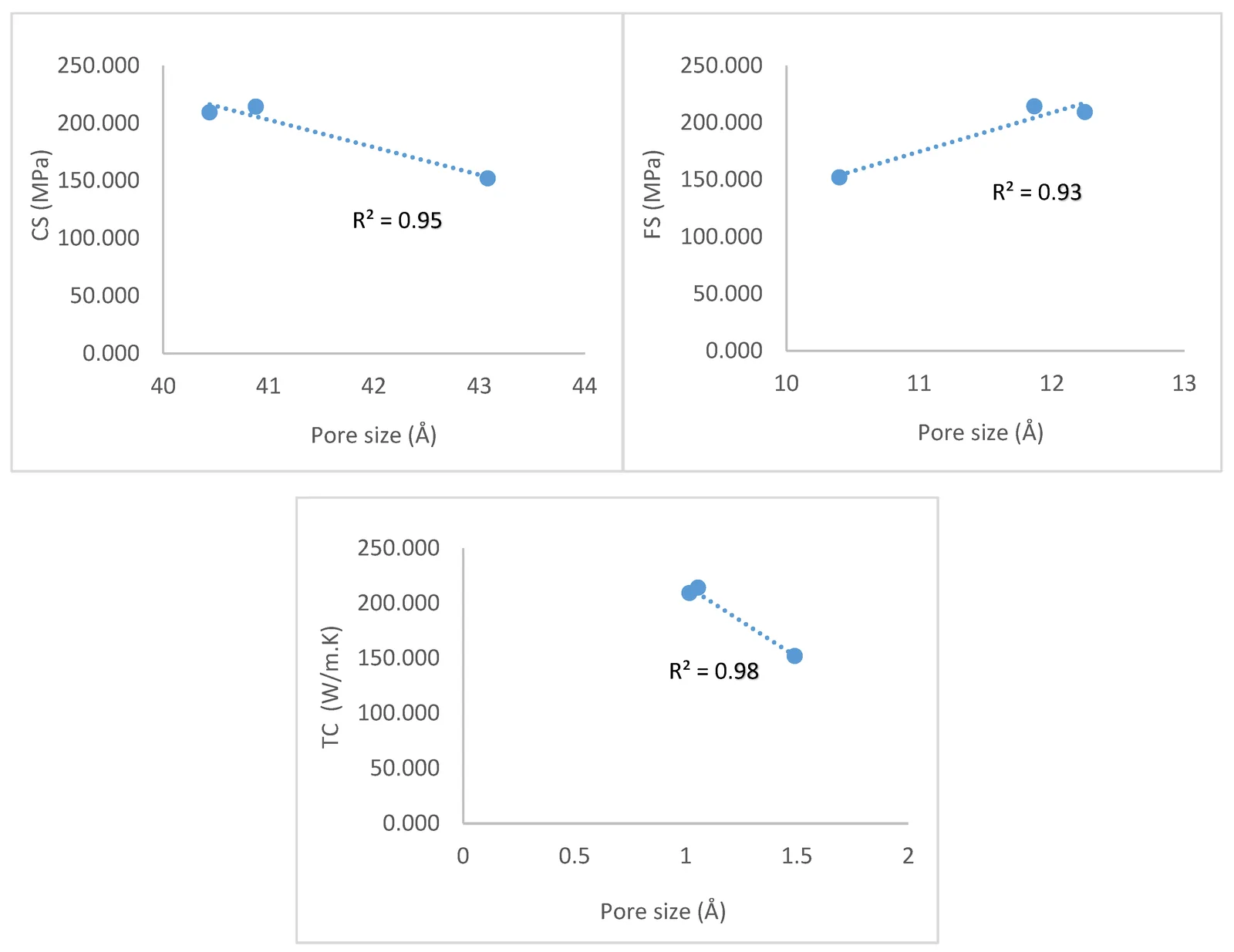
Correlations between the pore size and the mechanical and thermal properties of HybTP groups.

**Figure 13:**
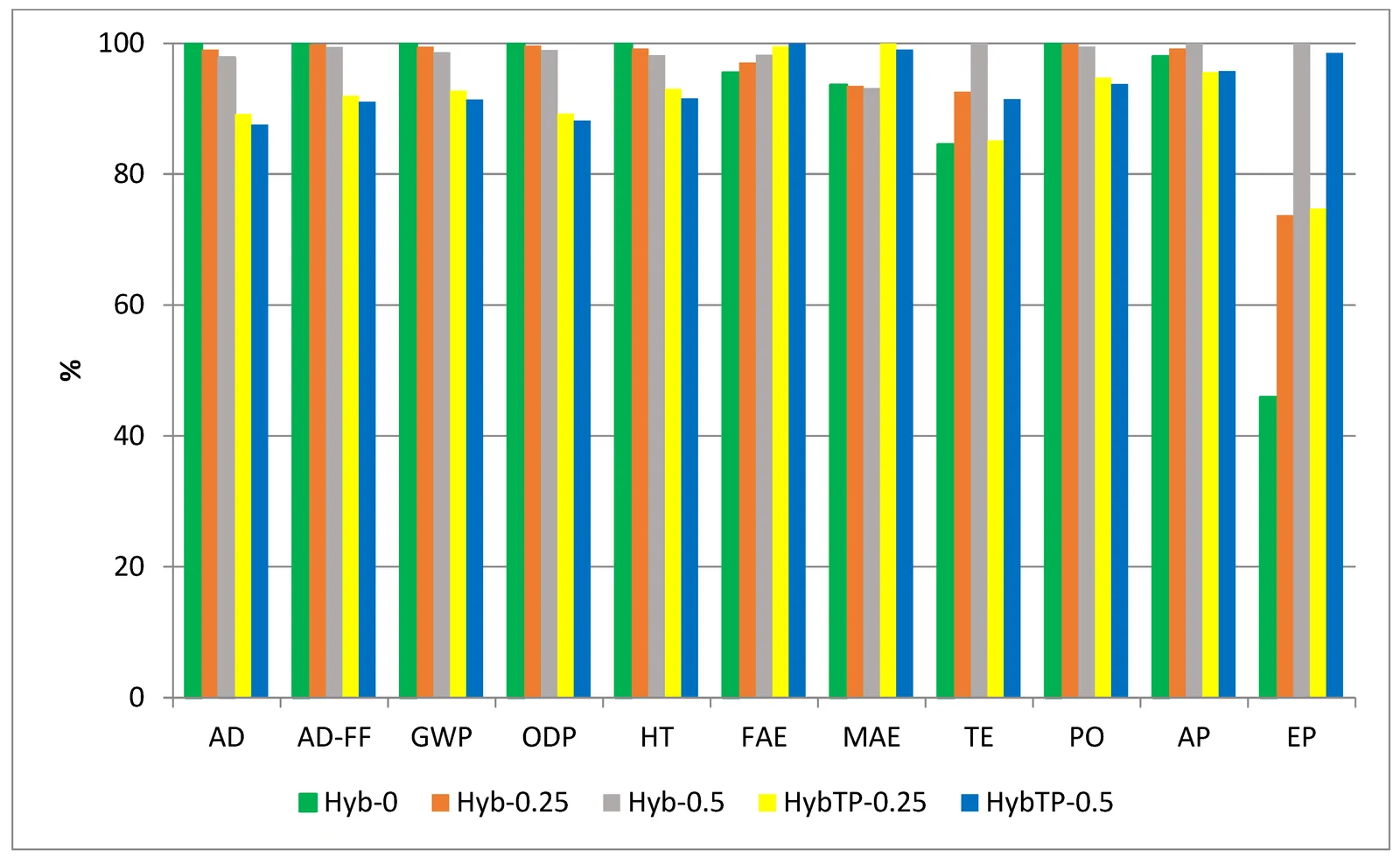
Comparison of the environmental impacts of mortar samples.

In Figure 11, the correlations between the mechanical and thermal characteristics of the Hyb group and the distribution of pore area were examined. The plotted data show consistent directional trends between the measured properties; however, owing to the limited number of observations, these relationships are considered exploratory and should not be interpreted as statistically validated correlations. This finding is consistent with the persistence of the predominance of fine pores smaller than 100 Å in fibers samples during BET analysis and the variation of the pore area distribution depending on the fiber content. Therefore, modifications in mechanical and thermal performance must to be assessed in addition to focusing on total pore volume but also by considering the relative impacts of various pore-size ranges.

In Figure 12, it is observed that the R^2^ values for the relationships between pore size and CS, FS, and TC in the HybTP group range from 0.93 to 0.98. This outcome suggests that modifications in mechanical and thermal performance correlate with the widening of the pore-size distribution identified in the BET analysis following the incorporation of WTRP. The detection of an expanded pore-size distribution together with diminished thermal conductivity values supports the microstructural evaluation that the pore structure could affect the continuity of heat conduction paths inside the solid phase. Nevertheless, pore size is not to be regarded as the exclusive factor influencing mechanical or thermal properties; the cumulative impacts of total pore volume, specific surface area, fiber–matrix and WTRP–matrix interface characteristics, along with pore-size distribution, must be considered.

Upon assessing the obtained correlations in combination with the mechanical, thermal, and microstructural findings, it becomes obvious that an optimal balance between mechanical and thermal performance, which is attributed to the incorporation of KCSFs and WTRP, is associated with changes in the pore structure. An increase of seed fiber content led to enhanced flexural strength and decreased thermal conductivity, whereas the incorporation of WTRP further diminished thermal conductivity but resulted a slight decline in compressive strength. The significant relationships observed between the pore characteristics determined via BET analysis and mechanical and thermal performance reveal that this behavior is associated not only with total pore volume but also with variations in pore size and distribution. Consequently, incorporating mechanical strength, thermal conductivity, and pore structure in the assessments designed to enhance mortars by utilizing kapok–cotton fibers and WTRP provides a comprehensive method for achieving the intended balance between mechanical and thermal performance.

### 3.6. Life Cycle Assessment (LCA)

The environmental performance of the Hyb-0, Hyb-0.25, Hyb-0.5, HybTP-0.25, and HybTP-0.5 mortars produced in this study was investigated utilizing life cycle assessment (LCA). To ensure the comparability of the mixtures, the functional unit was defined as 1 m^3^ of mortar, and the assessment was conducted within a cradle-to-gate system boundary covering the processes from raw material procurement to the completion of mortar production. Similarly, it is observed that a functional unit of 1 m^3^ and a cradle-to-gate approach are used in the comparison of mortar systems in recent LCA studies [72,73]. The environmental impact evaluation was performed utilizing the CML-IA Baseline methodology within SimaPro 9.5 software, including a total of 11 impact categories. The environmental impact assessment was performed utilizing the CML-IA Baseline method within SimaPro 9.5 software, including a total of 11 impact categories. The quantified impact categories included abiotic depletion (AD), abiotic depletion–fossil fuels (AD-FF), global warming potential (GWP), ozone depletion potential (ODP), human toxicity (HT), freshwater aquatic ecotoxicity (FAE), marine aquatic ecotoxicity (MAE), terrestrial ecotoxi city (TE), photochemical oxidation (PO), acidification potential (AP), and eutrophication potential (EP). Thus, the mixtures were evaluated within a broader environmental performance framework, encompassing not only carbon footprint but also resource consumption and ecotoxicity. The environmental impacts of the findings are displayed in Table 5.

An analysis of Table 5 reveals that the incorporation of kapok–cotton fibers and WTRP leads to different trends in the environmental impact categories. While minor numerical reductions were observed in the AD, AD-FF, GWP, ODP, HT, and PO categories for the Hyb-0.25 and Hyb-0.5 mixtures containing only KCSFs compared to Hyb-0, this improvement became more pronounced with the incorporation of WTRP into the mixture. Particularly for the HybTP-0.5 mixture, the AD, AD-FF, GWP, ODP, HT, PO and AP values decreased by approximately 12.1%, 8.9%, 8.5%, 11.9%, 8.7%, 6.3% and 2.5% respectively, compared to the reference. It should be noted that the environmental reductions observed for the WTRP-containing mixtures are influenced not only by material substitution but also by the mass of constituent materials required to produce the 1 m^3^ functional unit. Because the mixtures differ in density, conversion of the laboratory-scale proportions to a volumetric functional unit results in different material quantities per cubic meter, including a lower cement content for the lower-density WTRP-containing mixtures. Since cement is a major contributor to several environmental impact categories, this density-related reduction in cement demand contributes to the lower impacts observed for HybTP mixtures. Therefore, the reported environmental improvements should be interpreted as the combined consequence of mixture composition and density-based scaling to 1 m^3^, rather than as an effect of WTRP substitution alone. Moreover, the 1 m^3^ functional unit provides a volumetric comparison and does not normalize the mixtures for differences in mechanical strength or thermal performance. To further account for mechanical performance, the GWP values were also considered relative to the 28-day compressive strength. The resulting values were approximately 14.44 kg CO_2_-eq/(m^3^·MPa) for Hyb-0 and 14.07 kg CO_2_-eq/(m^3^·MPa) for HybTP-0.5, corresponding to a reduction of approximately 2.6%. This performance-normalized comparison indicates that the environmental advantage of HybTP-0.5 is substantially smaller when compressive strength is taken into account than when the mixtures are compared solely on a volumetric basis. Therefore, the environmental benefits should be interpreted in relation to the functional performance required for the intended application. No formal uncertainty analysis was performed in the present LCA. Therefore, relatively small differences between mixtures, particularly those within a few percentage points, should be interpreted as numerical trends rather than as definitive environmental improvements. Greater emphasis is consequently placed on the overall pattern across impact categories and on changes of larger magnitude. The baseline inventory explicitly includes the long-distance transportation of kapok fiber from the Philippines to Türkiye, including both maritime and road transportation stages. However, sensitivity analyses considering alternative transportation distances and energy structures were not performed in the present study; therefore, these parameters should be examined in future LCA studies to evaluate their influence on the environmental results. The present LCA was limited to a cradle-to-gate system boundary; therefore, potential use-phase energy effects associated with the reduced thermal conductivity of the modified mortars were not included in the environmental assessment. Moreover, although KCSF and WTRP incorporation reduced thermal conductivity relative to the reference mortar, the measured values should be interpreted as an improvement in the thermal performance of the mortar rather than as indicating that the material itself functions as a thermal insulation material. A cradle-to-grave assessment considering the use phase would be required to determine whether the observed reduction in thermal conductivity provides meaningful operational energy benefits in a building application.

The results therefore indicate that the WTRP-containing mixtures exhibited lower impacts in several categories, including resource utilization, climate change, stratospheric ozone depletion, and human toxicity. Conversely, the improvement in environmental performance has not progressed uniformly across all categories. Increases were observed in the FAE, MAE, TE, and especially EP categories compared to the reference mixture, and this trend became more pronounced in certain mixtures as the fiber content increased. Therefore, the use of bio-based KCSFs and waste-derived WTRP did not simultaneously reduce environmental impacts across all categories; instead, it led to environmental trade-offs between different impact categories. To provide a visual comparison of the different environmental trends identified among the mixtures, the outcomes for impact categories are illustrated in Figure 13.

**Figure 14:**
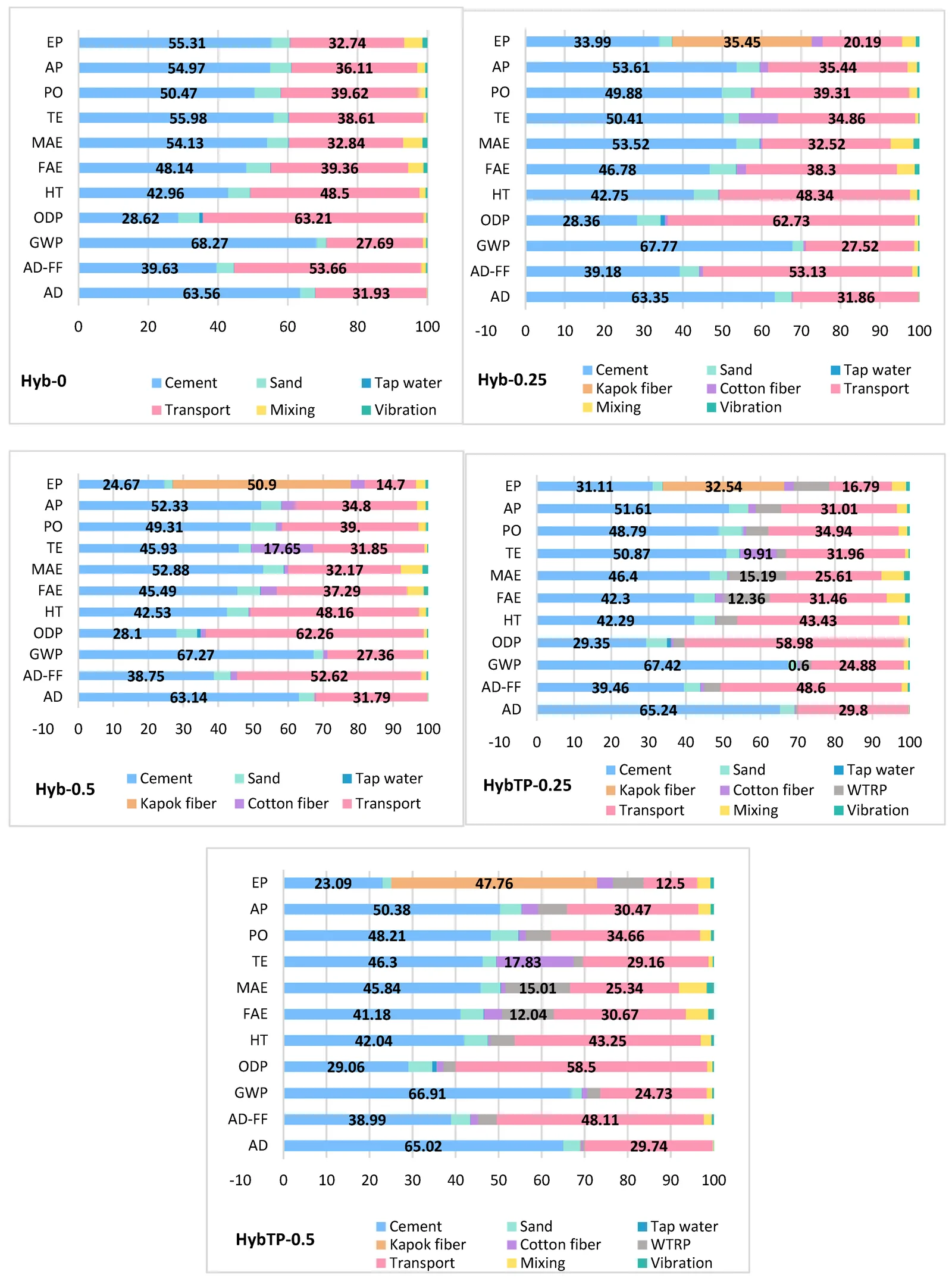
Relative contributions of raw material, transportation, and production processes to environmental impact categories in mortar mixtures.

Figure 13 visually supports the trends identified in Table 3 and clearly demonstrates the reduction in the AD, AD-FF, GWP, ODP, HT, PO and AP categories, particularly in mixtures containing WTRP. In contrast, different trends among the FAE, MAE, TE, and EP categories suggest that the effect of mixture composition on environmental impacts depends on the category. To analyze in more detail the life cycle inputs involved for these changes, the respective impacts of raw materials, transportation and production processes on environmental impact categories were assessed for each mortar mixture, with the findings illustrated in Figure 14.

The contribution analysis illustrated in Figure 14 reveals that the environmental impacts of the mortars are not evenly distributed among components and life cycle processes, and that the dominant sources of contribution vary depending on the impact category. In the reference Hyb-0 mixture, cement production and raw material transport have been identified as the primary sources of contribution across many impact categories. While the relative contributions of cement to the AD and GWP categories were approximately 63.6% and 68.3%, respectively, the contributions of transportation to the AD-FF and ODP categories reached 53.7% and 63.2%, respectively. In contrast, the relative contribution of electricity consumed for mixing and vibration remained more limited. The high contribution of cement, particularly to GWP, is related to the energy-intensive nature of clinker production as well as the process related CO_2_ emissions occurring during the calcination of the raw material. The impacts resulting from transportation depend on the quantity of material transported, the transportation method, and the supply distance. This research indicates that the road transport of natural sand across a distance of 610 km represents a significant contributor to the environmental impact related to transportation. The decisive role of cement production and raw material transport regarding environmental impacts is consistent with the findings of previous LCA studies that evaluated various binder and transport scenarios using a cradle-to-gate approach [74].

The incorporation of kapok and cotton fibers into the Hyb-0.25 and Hyb-0.5 mixtures caused a significantly change in the environmental contribution profile, particularly in the TE and EP categories. The relative contribution of kapok fiber in the EP for the Hyb-0.25 mixture was 35.4%, which increased to 50.9% in the Hyb-0.5 mixture with an increased fiber content. Similarly, the contribution of cotton fiber to TE increased from 9.8% in Hyb-0.25 to 17.6% in Hyb-0.5. This trend was also maintained in mixtures containing WTRP; the contribution of kapok fiber to EP was calculated as 32.5% and 47.8% for HybTP-0.25 and HybTP-0.5, respectively, while the contribution of cotton fiber to TE was calculated as 9.9% and 17.8%, respectively. Therefore, an increase in the fiber content ratio enhanced the relative contribution of kapok fiber to EP, and that of cotton fiber to TE, in particular.

This finding indicates that the fact that a bio-based raw material is of renewable origin should not be interpreted as automatically leading to improvements across all environmental impact categories. Recent studies evaluating mortars containing natural fibers using life cycle assessment (LCA) have demonstrated the necessity of assessing fiber usage based on environmental indicators alongside mechanical and thermal performance [73].

A significant observation in the TE category is the minor adverse relative contributions linked to kapok fiber. The contribution of kapok fiber to TE was calculated as approximately −0.04%, −0.07%, −0.04%, and −0.08% for the Hyb-0.25, Hyb-0.5, HybTP-0.25 and HybTP-0.5 mixtures, respectively. Nevertheless, these adverse contributions are minimal in size and do not indicate that the aggregate TE effect of the mixtures is negative; the total TE values were persistently positive for every mixture. Therefore, the negative values in question should be evaluated not as an environmental gain independent of the overall environmental performance, but as the relative contribution of the kapok production process within the life cycle inventory used to the TE category. Furthermore, although road and maritime transportation were included in the model for the sourcing of kapok fiber from the Philippines, fiber production and transportation are defined as distinct life cycle processes; therefore, it is inappropriate to attribute changes in TE and EP solely to the long transport distance.

The incorporation of WTRP into the system resulted in a notable change in the attribution of contributions, especially with the ecotoxicity categories. In the HybTP-0.25 and HybTP-0.5 mixtures, the relative contribution of WTRP to FAE was determined to be 12.4% and 12.0%, respectively, while its contribution to MAE was 15.2% and 15.0%. In contrast, while the contribution of WTRP to GWP remained at approximately 3% in both mixtures, its contribution to EP was 9.6% and 7.1% for HybTP-0.25 and HybTP-0.5, respectively. These results demonstrate that the life cycle impact of WTRP is category-specific; therefore, its direct contribution which is limited in terms of GWP cannot be generalized to other environmental impacts such as FAE, MAE or EP. However, the reduction in the amounts of cement and natural sand per unit volume in mixtures containing WTRP is consistent with the total reductions in the AD, AD-FF, GWP, ODP, HT, PO, and AP categories specified in Table 5.

In the LCA methodology, the environmental impacts from the prior life cycle of the waste tire were not explicitly assigned to the WTRP; rather, the energy consumption (240 kWh/t) associated with the processes of shredding, crushing, grinding, granulation, and size classification in WTRP production was incorporated within the system boundaries. In line with this modeling approach, the environmental performance associated with WTRP utilization has been addressed by considering not only the valorization of a waste material but also the combined assessment of changes in conventional raw material usage and the environmental impacts arising from recovery, energy and transportation processes. Accordingly, the extent of the environmental advantage obtained from utilizing a waste-derived raw material is influenced less by the actual reuse of the waste and more by the balance between the volume of traditional raw materials replaced and the resources—such as processing, energy, and transportation—necessary to transform the waste into a functional raw material. This approach is also consistent with recent LCA findings indicating that the environmental performance of mortars containing alternative or waste-derived raw materials can vary depending on mix composition, substitution level, production processes, and transportation conditions [72].

When the LCA findings were evaluated alongside the mechanical and thermal performance results, a remarkable simultaneous change was observed in terms of environmental and functional performance in the mixtures containing WTRP. Particularly in the HybTP-0.5 mixture, compared to the Hyb-0 reference, thermal conductivity decreased by 31.74% and GWP dropped by approximately 8.5%; meanwhile, flexural strength increased by 17.79%, and the reduction in compressive strength was limited to 6.13%. Thus, the combined use of WTRP and kapok–cotton fibers enabled the simultaneous reduction of thermal conductivity and global warming potential, while also allowing for improved flexural performance. However, the increases observed in the FAE, MAE, TE, and especially EP categories demonstrate that environmental gains cannot be generalized across all impact categories and that environmental trade-offs must be considered in sustainability assessments. In summary, the results indicate that the incorporation of kapok–cotton fibers and WTRP in cementitious mortars enhances thermal insulation and flexural strength, facilitates waste recycling, and meanwhile diminishes several life cycle environmental effects, especially global warming potential. This comprehensive performance illustrates that the KCSFs–WTRP hybrid mortar system signifies a potential material design strategy for creating more sustainable cement-based mortars that simultaneously address mechanical, thermal and environmental criteria.

## 4. Conclusions

In this study, the effects of KCSFs and WTRP on the mechanical, thermal, microstructural and life cycle-based environmental performance of Portland cement-based mortars were comprehensively evaluated. The fundamental results obtained are summarized below:

• The use of KCSFs resulted in significant improvements in flexural and thermal insulation performance, alongside a limited reduction in compressive strength. Compared to the Hyb-0 reference, mixtures containing only KCSFs showed a 2.16–2.79% decrease in 28-day compressive strength, while flexural strength increased by 14.81–18.65% and thermal conductivity decreased by 16.78–24.16%.
• The reduction in thermal conductivity became more pronounced with the incorporation of WTRP. The lowest thermal conductivity value, 1.017 W/mK, was achieved with the HybTP-0.5 mixture, representing a 31.74% reduction compared to the reference. In the same mixture, flexural strength increased by 17.79%, while the reduction in compressive strength was limited to 6.13%. The 28-day compressive strengths ranged from 40.44 MPa for HybTP-0.5 to 43.08 MPa for Hyb-0, indicating that all investigated mixtures maintained compressive strengths above 40 MPa under the present experimental conditions.
• While SEM analyses revealed fiber–matrix interactions supporting the crack-bridging behavior of KCSFs within the matrix, more heterogeneous matrix regions, localized voids, and interfacial discontinuities were observed in samples containing WTRP. These microstructural characteristics were found to be consistent with the increase in flexural strength and the limited decrease in compressive strength.
• XRD analyses primarily identified quartz (Q), poorly crystalline C–S–H-based hydration products, and portlandite (CH). The incorporation of KCSFs and WTRP did not lead to the formation of any new crystalline phases; the observed differences were attributed to changes in the relative distribution of existing crystalline phases and poorly crystalline hydration products. This result is consistent with KCSFs and WTRP functioning as reinforcement and replacement components rather than as reactive mineral constituents that form new crystalline hydration products.
• BET analyses revealed that the addition of KCSFs and WTRP changed the surface area, total pore volume, and pore-size distribution. While the dominance of fine pores within the total pore area was maintained in fiber-containing mixtures, it was determined that the addition of WTRP could lead to an increase in total pore volume despite a reduction in BET specific surface area. This variation was attributed to the redistribution of pore area across a wider range of pore diameters and was found to be consistent with the interfacial and microstructural changes observed via SEM.
• LCA results demonstrated that the environmental impacts associated with the use of KCSFs and WTRP varied depending on the impact category. Specifically, for the HybTP-0.5 mixture, values for AD, AD-FF, GWP, ODP, HT, PO, and AP decreased by approximately 12.1%, 8.9%, 8.5%, 11.9%, 8.7%, 6.3%, and 2.5%, respectively, compared to the reference. Conversely, increases were observed in the FAE, MAE, TE, and EP categories, with TE and EP increasing by up to approximately 19% and 118%, respectively, compared with the reference mixture. These substantial trade-offs demonstrate that environmental gains cannot be generalized across all impact categories and that the investigated mixtures should not be interpreted as providing uniformly improved environmental performance.
• When mechanical, thermal, and environmental outcomes are evaluated collectively, the HybTP-0.5 mixture demonstrated a remarkable balance of performance. Compared to Hyb-0, flexural strength increased by 17.79%, while thermal conductivity and GWP decreased by 31.74% and approximately 8.5%, respectively; the reduction in compressive strength was limited to 6.13%. This comprehensive result suggests that the synergistic application of kapok–cotton fibers and WTRP offers a promising approach for generating more sustainable cement-based mortars that effectively enhance thermal insulation, mechanical properties, and life cycle environmental performance.

Overall, this study has presented a material design approach that while maintaining the traditional Portland cement-based mortar structure and practical production method comprehensively focuses on the integration of KCSFs and WTRP concerning mechanical, thermal, and life-cycle-based environmental performance, without requiring additional activation processes such as alkali activation. Future studies incorporating kapok-only, cotton-only, and WTRP-only control mixtures, together with different kapok/cotton ratios and WTRP levels, would enable the individual and interaction effects of these constituents to be quantitatively distinguished. Long-term durability was not investigated in the present study and therefore remains an important limitation when considering the practical engineering applicability of the investigated mortars. Future studies should evaluate drying shrinkage, freeze–thaw resistance, carbonation resistance, and chloride ion penetration, as well as possible long-term changes in the fiber–matrix and WTRP–matrix interfaces, dimensional stability, and mechanical and thermal performance. In addition, investigating long-term durability characteristics and supplementing BET analysis with methods such as MIP and micro-CT will contribute to a multiscale assessment of the relationships between pore structure and performance. Complementary quantitative phase analysis using Rietveld refinement and/or thermogravimetric analysis (TGA) would also enable a more rigorous evaluation of changes in hydration-product contents and their relationship with mechanical performance. Furthermore, investigating the approach based on the combined use of KCSFs with WTRP in low-clinker and hybrid binder systems will enable the assessment of the proposed material design’s applicability to different binder systems. Expanding the scope of the LCA to include the use and end-of-life stages will also enable a more comprehensive assessment of environmental performance. In this respect, the study provides a scientific basis for a sustainable and viable material design approach based on integrating renewable natural fibers and waste-derived WTRP into conventional Portland cement-based mortars that simultaneously addresses the maintenance of mechanical performance and the improvement of thermal and life-cycle-based environmental performance.

## Figures and Tables

**Table 1:** Mixture mortar designs and proportions.

Mix	KP (g)	CO (g)	Cement (g)	Sand (g)	Water (mL)	WTRP (g)
Hyb-0	-	-	450	1350	225	-
Hyb-0.25	1.125	1.125	450	1350	225	-
Hyb-0.5	2.225	2.225	450	1350	225	-
HybTP-0.25	1.125	1.125	450	1215	225	135
HybTP-0.5	2.225	2.225	450	1215	225	135

**Table 2:** Life cycle inventory of raw materials for the production of 1 m^3^ of mortar.

Mix	KP (kg/m^3^)	CO (kg/m^3^)	Cement (kg/m^3^)	Sand (kg/m^3^)	Water (kg/m^3^)	WTRP (kg/m^3^)	Mortar Density (kg/m^3^)
Hyb-0	0.00	0.00	466.67	1400.00	233.33	0.00	2100
Hyb-0.25	1.15	1.15	460.60	1381.80	230.30	0.00	2075
Hyb-0.5	2.24	2.24	453.45	1360.34	226.72	0.00	2045
HybTP-0.25	1.07	1.07	427.30	1153.72	213.65	128.19	1925
HybTP-0.5	2.07	2.07	417.97	1128.52	208.99	125.39	1885

**Table 3:** The supplier locations, transportation methods and distances utilized in the life cycle inventory of raw materials.

Raw Material	Supplier Locations	Transportation Methods	Transport Distances (km)
Portland cement	Afyon Cement	Road transportation	115
Rilem Cembureau type sand	Trakya Limak Cement Factory	Road transportation	610
Waste Tire Rubber Powder	Katek Ağaoğlu Rubber Technologies, Uşak	Road transportation	15
Kapok Fiber	Philippines	Road transportation—Within Philippines	100
Kapok Fiber	Philippines → Türkiye	Maritime transport—Container ship	11.000
Kapok Fiber	Türkiye	Road transportation—Within Türkiye	260
Pamuk Fiber	Aydın, Söke	Road transportation	266

**Table 4:** Electrical energy inventory inputs for the production of 1 m^3^ of mortar (kwh).

Mix	Mixer Energy	Vibration Energy	Total Energy
**Hyb-0**	10.41	2.60	13.01
**Hyb-0.25**	11.00	2.75	13.75
**Hyb-0.5**	11.70	2.90	14.60
**HybTP-0.25**	12.20	3.00	15.20
**HybTP-0.5**	13.00	3.20	16.20

**Table 5:** Environmental impact assessment results of mortar mixtures based on a 1 m^3^ functional unit.

Impact Category	Unit	Hyb-0	Hyb-0.25	Hyb-0.5	HybTP-0.25	HybTP-0.5
**AD**	kg Sb eq	1.74E-03	1.73E-03	1.71E-03	1.56E-03	1.53E-03
**AD-FF**	MJ	4.50E+03	4.50E+03	4.48E+03	4.14E+03	4.10E+03
**GWP**	kg CO_2_ eq	6.22E+02	6.19E+02	6.14E+02	5.77E+02	5.69E+02
**ODP**	kg CFC-11 eq	3.61E-06	3.60E-06	3.57E-06	3.22E-06	3.18E-06
**HT**	kg 1,4-DB eq	1.73E+02	1.71E+02	1.69E+02	1.61E+02	1.58E+02
**FAE**	kg 1,4-DB eq	9.17E+01	9.32E+01	9.43E+01	9.56E+01	9.60E+01
**MAE**	kg 1,4-DB eq	2.20E+05	2.19E+05	2.19E+05	2.35E+05	2.32E+05
**TE**	kg 1,4-DB eq	1.55E+00	1.70E+00	1.84E+00	1.57E+00	1.68E+00
**PO**	kg C_2_H_4_ eq	6.94E-02	6.93E-02	6.90E-02	6.57E-02	6.50E-02
**AP**	kg SO_2_ eq	1.58E+00	1.60E+00	1.61E+00	1.54E+00	1.54E+00
**EP**	kg PO_4_- eq	4.39E-01	7.05E-01	9.57E-01	7.15E-01	9.42E-01

## Data Availability

The original contributions presented in this study are included in the article. Further inquiries can be directed to the corresponding author.

## Abbreviations

The following abbreviations are used in this manuscript:

KCSFs	Kapok–cotton seed fibers
WTRP	Waste tire rubber powder
KP	Kapok fiber
CO	Cotton fiber
SEM	Scanning electron microscope
XRD	X-ray diffraction
TG-DSC	Thermogravimetry and differentıal scanning calorimetry
ITZ	Interface transition zone
LCA	Life cycle assessment
LCIA	Life cycle impact assessment
AD	Abiotic depletion
AD-FF	Abiotic depletion–fossil fuels
GWP	Global warming potential
ODP	Ozone depletion potential
HT	Human toxicity
FAE	Freshwater aquatic ecotoxicity
MAE	Marine aquatic ecotoxicity
TE	Terrestrial ecotoxicity
CS	Compressive strength
FS	Flexural strength
TC	Thermal conductivity
